# Development of Uniform Porous Carbons From Polycarbazole Phthalonitriles as Durable CO_2_ Adsorbent and Supercapacitor Electrodes

**DOI:** 10.3389/fchem.2022.879815

**Published:** 2022-04-25

**Authors:** Ghadeer Thani Alenezi, Narendran Rajendran, Ahmed Abdel Nazeer, Saad Makhseed

**Affiliations:** ^1^ Department of Chemistry, Faculty of Science, Kuwait University, Kuwait City, Kuwait; ^2^ Petroleum Refining and Petrochemicals Research Center, College of Engineering and Petroleum, Kuwait University, Kuwait City, Kuwait

**Keywords:** network polymer, pyrolysis, porous carbon, CO_2_ uptake, energy storage

## Abstract

Advances in new porous materials have recognized great consideration in CO_2_ capture and electrochemical energy storage (EES) applications. In this study, we reported a synthesis of two nitrogen-enriched KOH-activated porous carbons prepared from polycarbazole phthalonitrile networks through direct pyrolysis protocol. The highest specific surface area of the carbon material prepared by pyrolysis of p-4CzPN polymer reaches 1,279 m^2^ g^−1^. Due to the highly rigid and reticular structure of the precursor, the obtained c-4CzPN–KOH carbon material exhibits high surface area, uniform porosity, and shows excellent CO_2_ capture performance of 19.5 wt% at 0°C. Moreover, the attained porous carbon c-4CzPN–KOH showed high energy storage capacities of up to 451 F g^−1^ in aqueous electrolytes containing 6.0 M KOH at a current density of 1 A g^-1^. The prepared carbon material also exhibits excellent charge/discharge cycle stability and retains 95.9% capacity after 2000 cycles, indicating promising electrode materials for supercapacitors.

## Introduction

One of the daily challenges many developing countries facing in recent times is environmental degradation resulting from the critical emission of CO_2_ into the atmosphere (Bhattacharyya et al., 2021). It has been reported in many countries that the exemplary activities of large industries have led to an increase in carbon dioxide emissions into the atmosphere (Aslam et al., 2021). Presently, humanity’s basic energy needs are met by burning coal, oil, and natural gas, which are considered the focal anthropogenic sources of CO_2_ emissions and threaten the sustainable environment through global warming effects (Bonneuil et al., 2021). Thus, the most innovative technologies are needed to control the concentration of carbon dioxide in the atmosphere. Carbon capture and storage (CCS) is one of the propitious technologies that could be used to diminish CO_2_ emissions from the point sources of strong CO_2_ emissions. CCS involves capturing and transporting CO_2_ to the storage site. The main challenge of the whole process is the capture of CO_2_, as the costs allied with this process are expensive and make CCS difficult to apply in the commercial sector (Gür, 2022). Porous materials with pore sizes in the nanometer range could play an important role in CCS as they not only afford high adsorption competence and good selectivity but also have the benefit of low cost (Singh et al., 2020).

Currently, substantial research advances have been reported in the field of micro- and mesoporous materials for CO_2_ capture applications, mainly focusing on materials termed zeolite–imidazole frameworks (ZIFs), metal–organic frameworks (MOFs), activated carbon, and porous organic polymers and polymer membranes (Farmahini et al., 2021). Many factors play a role in CO_2_ capture applications, including surface area, pore volume, pore size, density, and functional groups that contribute to CO_2_ adsorption (Siegelman et al., 2021). Recent studies on the adsorption of CO_2_ with a variety of materials with tunable pore diameters suggest that the pore size of the adsorbent is a crucial factor, especially materials with micropores are well suited for low-pressure CO_2_ uptake; hence, they are very beneficial for post-combustion CO_2_ capture (Wang X. et al., 2021). In addition, nanoporous carbons provide ease of handling of their microporous- or mesoporous-sized pore structure, which can be appropriate for carbon dioxide adsorption under low- and high-pressure atmosphere (Deng et al., 2021). The accumulation of basic species on the surface of the porous carbons generally supports the material for increased CO_2_ adsorption through acid–base interactions. The enrichment of heteroatoms, especially N atoms, could adjust the electronic state of the hydrogen atoms present in the -CH and -NH groups of the carbon material and accommodate the formation of hydrogen bonds with the O atom of the CO_2_ molecule (Wang et al., 2020). Many reports are available for the development of porous carbon for CO_2_ uptake and storage applications. For instance, Shi et al. (2022) reported a series of heteroatom-doped porous carbons developed from the bio-based benzoxazine resin by a pyrolysis method. The synthesized carbon materials possess enriched heteroatom content, high surface area (1810 m^2^ g^−1^), and an exceptional CO_2_ uptake capacity of 6.78 mmol g^−1^ at 0°C. In addition, the carbon material showed a superior performance as an anode with specific capacities of 310 and 221 Fg^−1^ in 6 M KOH at a current density of 1 Ag^−1^. Similarly, Chen et al. (2022) proposed a strategy for the synthesis of porous carbons by chemical activation with KOH using porous organic polymer precursors for CO_2_ capture. The prepared carbon exhibits an excellent BET surface area of 3,367 m^2^ g^−1^ and an extraordinary CO_2_ adsorption ability of 7.78 mmol g^−1^ at 0°C. The ideal synthesis of activated carbons depends on crucial key factors such as precursors, activating agents, and the optimized protocol. Based on the available reports, KOH is considered the most effective activating agent when preparing carbon using the pyrolysis method. This is because KOH can suppress the formation of tar, stimulate the low temperature reaction, and help complete the pyrolysis process (Gao et al., 2020). There are three steps in KOH activation mechanisms that have been demonstrated. First, potassium penetrates the internal structure of the carbon lattice, then swells the space of the aromatic layer, and deforms the carbon layer to generate new pores. Later, it changes the electron density distribution of carbon atoms, thus producing more active reaction sites. Finally, it advances the wettability of the carbon surface and weakens the surface tension. Therefore, the role of metallic potassium vapor is exclusive and significant for the development of uniform pores during pyrolysis. Acidic or neutral activating agents fail to achieve this key factor, which could be the reason for the selection of KOH as an activating agent for the preparation of carbonaceous materials (Goel et al., 2021).

In addition, polycarbazole and its carbon materials have been widely studied as promising materials for dual applications such as gas adsorption and electrochemical energy storage (Bekkar et al., 2020; Nayana and Kandasubramanian, 2020). The development of electrode materials is due to their excellent hole transport properties, comparatively high specific capacitance, and outstanding atmospheric stability. Furthermore, in addition to their physical and electronic properties, such as surface morphology, thickness, and electrical conductivity, the internal resistance and durability directly affect supercapacitor performances (Vandeginste, 2022). For example, Wang et al. (2017) reported a polycarbazole-derived porous carbon with a high content of nitrogen, uniform pore size, and a large BET surface area of 1,280 m^2^ g^−1^, with a highly efficient CO_2_ capture of 20.4 wt% at 0°C. Moreover, the synthesized carbon materials show excellent electrochemical performance with a fast charge/discharge rate along with an excellent electrochemical capacity of 558 F g^−1^. Later, Ates and Uludag (2015) synthesized a poly (9H-carbazole-9-carbothioic dithioperoxyanhydride) film-based capacitor that showed a double-layer capacitance of 571 μF. Durantini et al. (2020) reported a Zn(II) porphyrin monomer modified with fully conjugated carbazole units by electrochemical polymerization. The polymer has excellent electronic properties due to the pseudocapacitance produced by the reversible redox processes of up to 277 F g^−1^. Duran et al. (2020) analyzed the supercapacitive performance of electrodeposited poly (carbazole) films with different supporting electrolytes and reported the best capacitance result of 133 F g^−1^ in the presence of lithium perchlorate. The present study deals with the synthesis of highly efficient carbonaceous materials from carbazole-tagged phthalonitrile network polymers for dual emerging environmental applications such as gas adsorption and energy storage. Here, we synthesized carbon materials (c-2CzPN–KOH and c-4CzPN–KOH) from microporous polycarbazole networks (p-2CzPN and p-4CzPN) using a KOH-activated pyrolysis method. All the synthesized carbons exhibit excellent BET surface area and CO_2_ uptake capacity. The complete capacitance studies of the resulting materials were investigated, which sheds light on the great potential of c-4CzPN–KOH as an electrode material for the high-performance supercapacitor; it exposed a specific capacitance of 451 F g^−1^ at 1.0 A g^−1^ with constant cycling performance in a three-electrode system, and it retains 95.9% capacitance after 2000 cycles at a current density of 2 A g^−1^. The output of the study provides a promising heteroatom-doped activated carbon with excellent gas uptake and electrochemical performance.

## Experimental Section

### Chemicals and Reagents

N, N-Dimethyl formamide, carbazole, tetrafluoroterephthalonitrile, dichloromethane, and methanol were obtained from Sigma-Aldrich. Cesium fluoride and 4,5-dichlorophthalonitrile were purchased from Alfa Aesar. Chloroform and ethanol were procured through Merck. Tetrahydrofuran (THF) was received from Thermo Fisher Scientific. De-ionized water was attained from the ELGA unit. The reactions were performed using clean, dried glassware under a N_2_ atmosphere. The chemicals procured were used without additional purification.

### Instrumentation

FT-IR spectra were performed to examine the functional groups of the materials using a JASCO FTIR 6300 instrument. ^1^H, ^13^C NMR, and CP-MAS analysis were acquired by using a Bruker AVANCE II 600 MHz instrument. HR (high-resolution) mass can be performed by using a GC-MS DFS Thermo instrument. PXRD (x-ray diffraction) patterns were recorded using a BRUKER D8 Advance diffractometer. TGA (thermogravimetric analysis) was achieved on a SHIMADZU DTG-60 thermal analyzer under N_2_ atmosphere. Differential scanning calorimetry (DSC) analysis was carried out using a NETZSCH 204 F1 Phoenix instrument. Scanning electron microscope (SEM) images with EDX can be obtained using a JEOL model JCM5700, and the materials were sampled by spraying the double-sided tape attached to a carbon stub and then sputter-coated with a thin film of gold. X-ray photoelectron spectra (XPS) were measured to study the elemental composition of the materials using a ESCALAB 250Xi XPS/UPS system. For ultraviolet photoelectron spectroscopy (UPS) analysis, the He(II) (21.21 eV) line is applied to the samples using a negative bias to shift the spectra from the spectrometer threshold. N_2_ and CO_2_ adsorption–desorption isotherms of the prepared materials were measured using a Micromeritics ASAP 2020 instrument. Nitrogen sorption isotherms were carried out at 77 K, and CO_2_ adsorption–desorption isotherms were recorded at 295 and 273 K, respectively. The specific surface area of the materials formed was calculated using the BET method. The pore-sized distribution of the materials was measured by the NLDFT method. High purity gases (99.999%) were used to perform static adsorption experiments. A Zetasizer Nano ZS instrument (Malvern, United Kingdom) was utilized to measure the particle size. The Raman spectrum was achieved using an InVia Renishaw Raman microscope, United Kingdom.

### Synthesis of c-2CzPN–KOH and c-4CzPN–KOH

To a solution of monomer (1.0 mmol, 500 mg) dissolved in CHCl_3_ (30 ml), three equivalents of calculated amount of FeCl_3_ (in terms of one carbazole and two active sites) were added and left at room temperature for 48 h. Then, the reaction mixture was filtered, and the soluble part was separated. The resulting precipitate was filtered off and purified by washing with deionized water, methanol, and THF. The resulting orange solid was dried under vacuum at 80°C for 12 h. The prepared polymers were mixed with potassium hydroxide (1:1 by weight ratio) and subjected to pyrolysis at 800°C under nitrogen (with a flow of 50 ml min^−1^) atmosphere and carbonized at 800°C for 30 min, yielding the porous carbon materials c-2CzPN–KOH and c-4CzPN–KOH. The carbon was further purified by refluxing with methanol and activation under vacuum at 120°C for 12 h.

### Electrochemical Measurements

An electrochemical potentiostat Gamry (Model Reference 3,000) was utilized to carry out all electrochemical experiments at room temperature. Carbon materials (c-2CzPN–KOH and c-4CzPN–KOH), polytetrafluoroethylene (PTFE), and carbon black were blended evenly in an 80:10:10 mass ratio. Then, 3.0 mg of the blend was applied onto nickel foam. All of the experiments were conducted in the 6 M KOH electrolyte using a standard three-electrode setup with the counter electrode (platinum wire) and reference electrode (Hg/HgO).

EIS tests were measured in the frequency range of 10 MHz–100 kHz, with a 10 mV AC amplitude. The following equation is used to calculate the specific capacitances obtained from GCD curves:

C=IΔt/mΔV, (1)

where C is the specific capacitance (F.g^−1^), I represents current (A), Δt is the discharge time (s), m is the mass of the coated active material (g), and ΔV is the potential window.

## Results and Discussion

### Synthesis of CzPN Polymers and Their Carbon Materials

**SCHEME 1:**
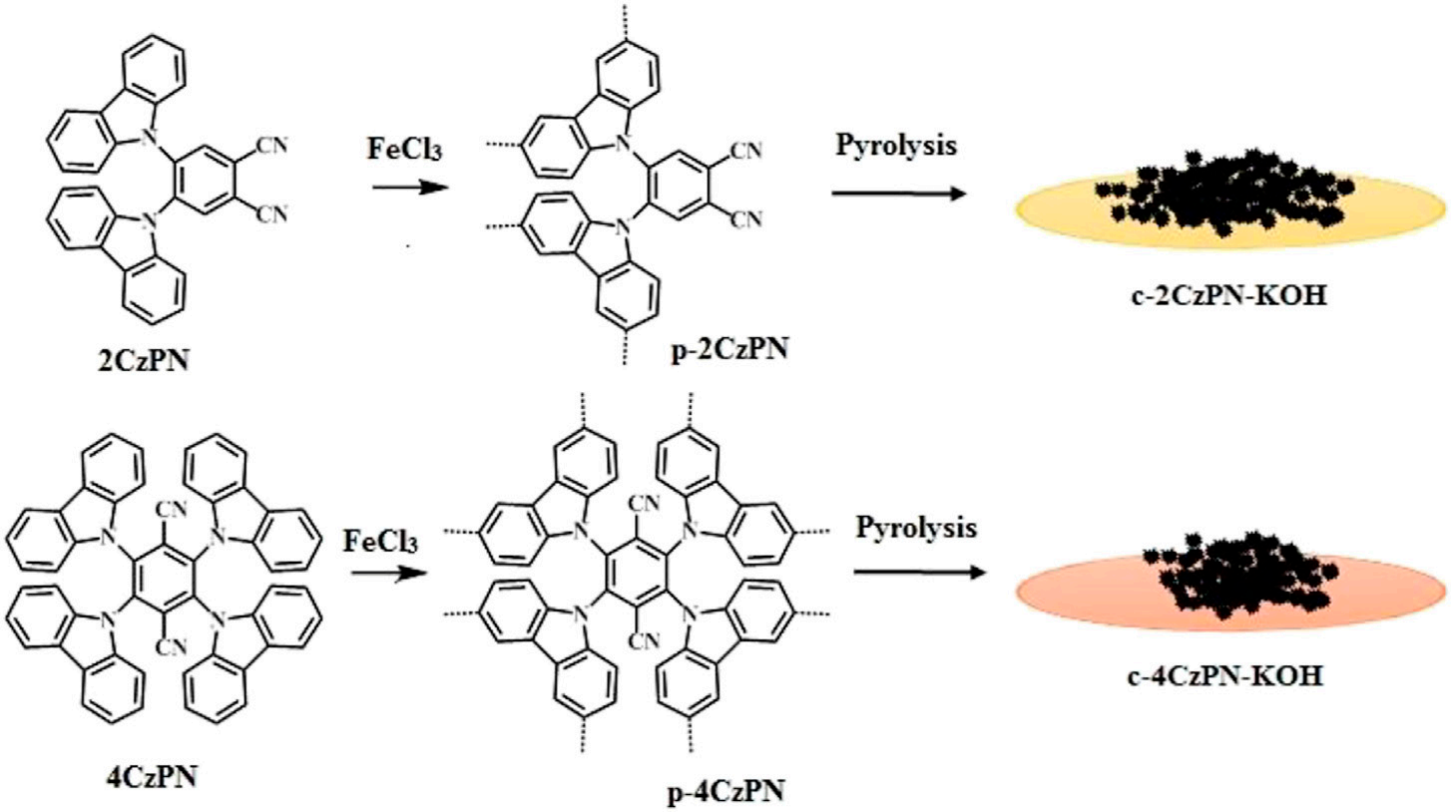
Synthesis of the network polymers p-2CzPN, p-4CzPN, and their carbon materials c-2CzPN-KOH and c-4CzPN-KOH.

The designed monomers (2CzPN and 4CzPN) were synthesized according to the reported protocol (Rajendran et al., 2020). The monomers 2CzPN and 4CzPN were tagged with two and four carbazole units synthesized using conventional nucleophilic substitution reaction. The two hypercrosslinked porous organic polymers p-2CzPN and p-4CzPN were prepared by an FeCl_3_-mediated oxidative polymerization method (Scheme 1). The crude polymeric materials were purified by refluxing with methanol, methanol/water, and THF to give yellow powder with good yield (>90%). In addition, the carbon materials were produced by a direct pyrolysis method (Fu et al., 2021). The prepared CzPN polymers were mixed with KOH and heated to 800°C under nitrogen atmosphere (with a N_2_ flow of 50 ml min^−1^) and pyrolyzed at 800°C to yield the carbon materials c-2CzPN–KOH and c-4CzPN–KOH, which were further purified by refluxing with methanol and activation at 120°C for 12 h. The purified carbon materials were subjected to characterization and other measurements.

### Characterization

The FT-IR spectroscopic analysis depicted that the nitrile C-N stretching frequency of 2CzPN and p-2CzPN was obtained at 2,234 cm^−1^ and 2,237 cm^−1^, respectively, which indicated that the functional C-N group was active after polymerization (Supplementary Figure S1A). In case of 4CzPN and p-4CzPN, the C-N stretching frequency was attained at 2,234 cm^−1^ and 2,235 cm^−1^, respectively (Supplementary Figure S1B). The C-N stretching vibration was clearly visible and there was a constant reduction found in the fingerprint region of both p-2CzPN and p-4CzPN; therefore, it can be concluded that the polymerization was suitably carried out without affecting the functionalities (Yu et al., 2018). Supplementary Figure S2 represents the FTIR spectrum of carbon materials. Both the carbon materials show a definite peak at 1,579 cm^−1^ representing the polyaromatic C=C stretching vibrations in sp^2^-hybridized carbons. The C-O stretching frequency obtained at 1,227–1,210 cm^−1^ is due to the use of the KOH activation method. The C-H stretching frequency was obtained as a broad range between 3,300 and 2,900 cm^−1^. FT-IR spectrum evidently confirmed the complete formation of carbon materials (Nagalakshmi et al., 2015). In order to find the purity of graphitization, Raman analysis was executed (Supplementary Figure S3). The D band obtained at 1,350 cm^−1^ is attributed to amorphous carbon, and the G band attained at 1,580 cm^−1^ is produced by tangential vibration of the ordered carbon atoms. The sharp clear peaks clearly indicate the purity of the pyrolyzed materials.

To confirm the structure of CzPN monomers, ^1^H and ^13^C NMR were performed. Both the monomers exhibit all of the fingerprint signals of the carbazole unit (Supplementary Figures S4, S8). The aromatic proton signal obtained at 8.83 ppm ascribed to the proton of a benzene ring belongs to the phthalonitrile moiety. This was further verified by ^13^C NMR, and all signals obtained were assigned to the monomer structure (Supplementary Figures S5, S9). The signal achieved at 115 ppm belongs to the nitrile carbon, and the signals in the range of 100–140 ppm were assigned to the phenyl carbons of the monomer structure. A cross-polarization magic angle spinning (CP-MAS) NMR spectrum was achieved for p-2CzPN and p-4CzPN polymers (with a spinning rate of 10 kHz, delay time of 5 s, and contact time of 2000 µ sec), and a signal acquired between 114 and 115 ppm was allotted to the -C≡N carbon attached to the benzene ring. In addition, the other chemical shifts in the range of 100–140 ppm were assigned to phenyl carbon atoms. The CP-MAS spectrum of p-2CzPN and p-4CzPN displayed carbon signals similar to those of the monomer (Supplementary Figures S7, S10) (Chen et al., 2012). In addition, the mass and purity of the CzPN monomers were inveterate as confirmed by HPLC and HRMS analysis. The HR-MS analysis of the prepared monomers clearly followed the calculated values, which helped to confirm the structure of the monomers (Supplementary Figures S11, S12). Furthermore, the purity of the monomers was examined using a high-performance liquid chromatography (HPLC) method. Both the prepared monomers exhibited a high purity of >99% by area (Supplementary Figures S13, S14).

The stability of the polymer mainly depends on the stable building blocks. For example, the DSC analysis of the monomers 2CzPN and 4CzPN showed a sharp melting point at 343.6°C and 497.2°C, respectively, which indicated that the synthesized monomers (2CzPN and 4CzPN) were highly stable even above 300°C (Supplementary Figure S15). Owing to the cross linking nature, polycarbazoles are thermally stable materials. To study the thermal stability of the materials, a TGA analysis was recorded for p-2CzPN and p-4CzPN polymers under nitrogen atmosphere (Supplementary Figure S16). TGA analysis shows that only 5% weight loss was observed up to 300°C, and the maximum weight loss of 40% attained at 600°C revealed that the prepared polymers p-2CzPN and p-4CzPN exhibit high stability due to the increasing substitution of carbazole entities (Li et al., 2017). PXRD patterns were performed to show the non-crystalline nature of the materials. p-2CzPN and p-4CzPN do not show any significant peaks, which clearly revealed their amorphous nature (Supplementary Figure S17). The PXRD spectrum of c-2CzPN–KOH and c-4CzPN–KOH offers a big broad amorphous carbon peak at about 20⁰ (002) and a localized graphitization peak at 44⁰ (100), along with silica substrate peaks (Supplementary Figure S18) (Rajendran et al., 2021). The PXRD analysis of the carbon materials clearly implicated the formation of carbon materials.

**FIGURE 1:**
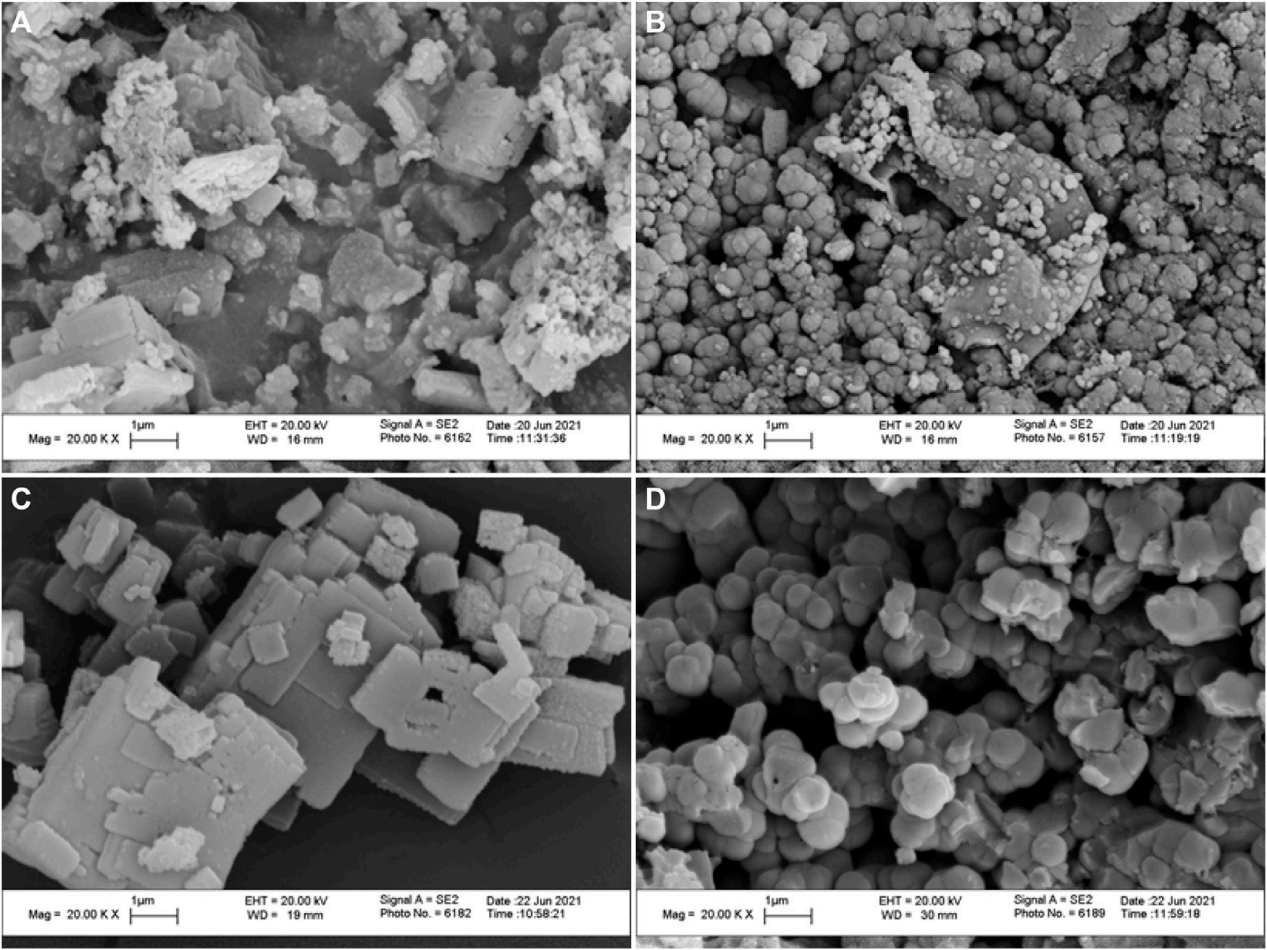
Scanning electron microscope images of **(A)** p-2CzPN, **(B)** c-2CzPN–KOH, **(C)** p-4CzPN, and **(D)** c-4CzPN–KOH.

The morphological investigations of the produced materials were carried out using a SEM (Figure 1). p-2CzPN showed different random sized particle distribution (Figure 1A), and p-4CzPN polymer was packed with diagonal sheets (Figure 1C). Furthermore, the synthesized carbon materials exhibited uniform spherical particles between 100 nm and 1 µm particle size distribution (Figures 1B,D). Moreover, the size of the carbon particles was verified by particle size analysis. The obtained carbon materials clearly show the particle size distribution between 0.3 nm and 1 µm (Supplementary Figure S19). The morphological and particle size analysis clearly confirmed that the pyrolysis was performed successfully with formation of uniform carbon materials (Vafaeinia et al., 2022). The elemental composition of polycarbazole phthalonitrile polymers and the carbon materials was studied by XPS analysis (Supplementary Figure S20). Both p-2CzPN and p-4CzPN displayed the acceptable percentages of C and N with traces of chloride contamination (Supplementary Table S1). The survey scan of the polymers indicated that there is no sign of iron between 708 and 710 eV, which indicates the purity of the polymers. In addition, the synthesized carbon materials show carbon atomic percentages of 89.1 and 90.2, respectively. Also, the nitrogen atomic percentage decreased significantly after pyrolysis. The XPS analysis revealed that the enriched C atoms and traces of O and N contents were produced in carbon materials. This could be due to the evaporation of heteroatoms; furthermore, potassium hydroxide reacted with C and N to form small gas molecules (H_2_O, CO_2_, CO, and oxidized nitrogen species NOx) due to dehydration, denitrogenation, and decarboxylation. The formation of chemical composition during pyrolysis was further evaluated by EDX measurements. The EDX spectra of the carbon materials evidently show the dominant proportions of carbon, which further supports the successful completion of the pyrolysis process (Supplementary Figures S21, S22). The elemental analysis study described that the carbonization was performed successfully (Kamran and Park, 2020).

### Textural Properties

**FIGURE 2:**
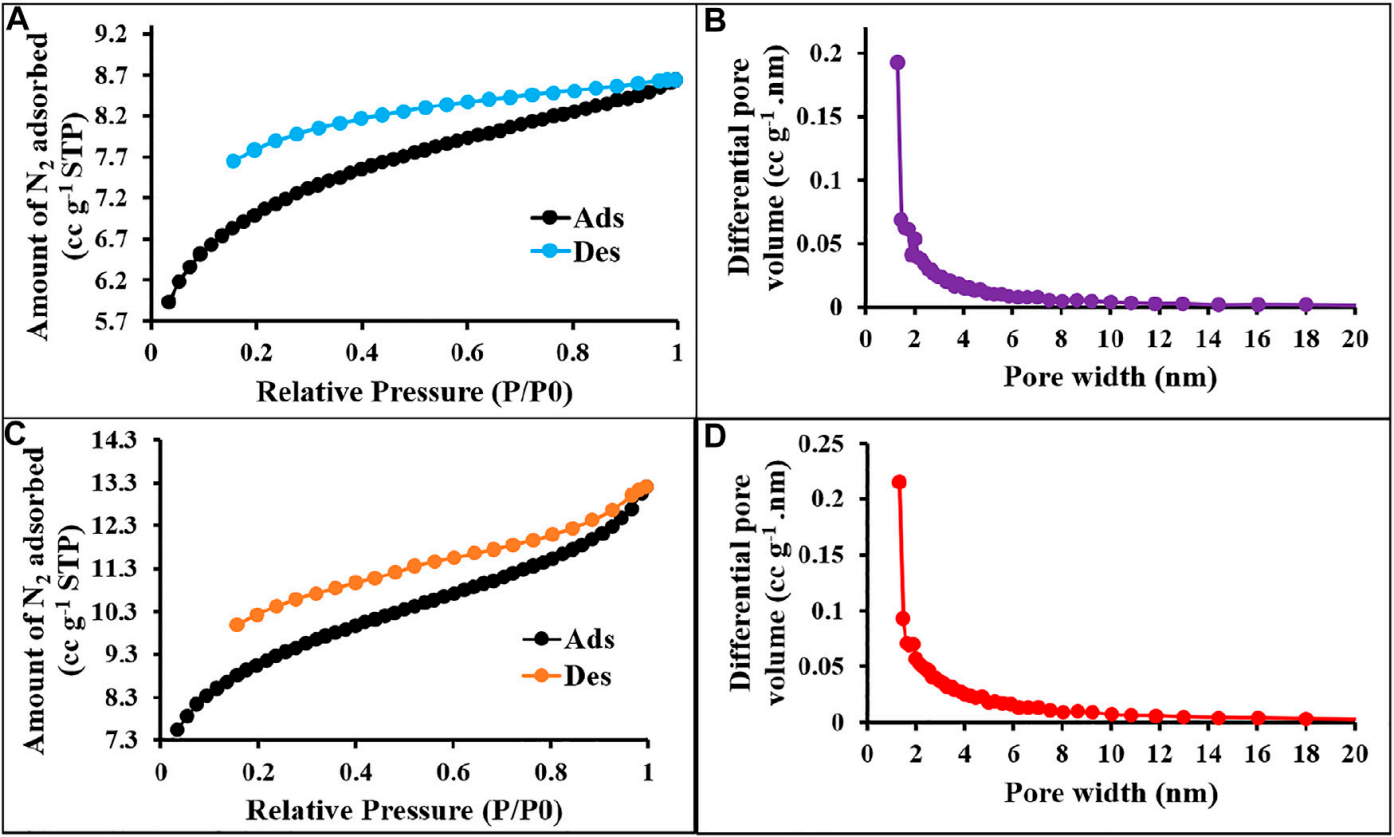
N_2_ sorption–desorption isotherms and pore size distribution curves of p-2CzPN **(A,B)** and p-4CzPN **(C,D)** at 77 K.

**FIGURE 3:**
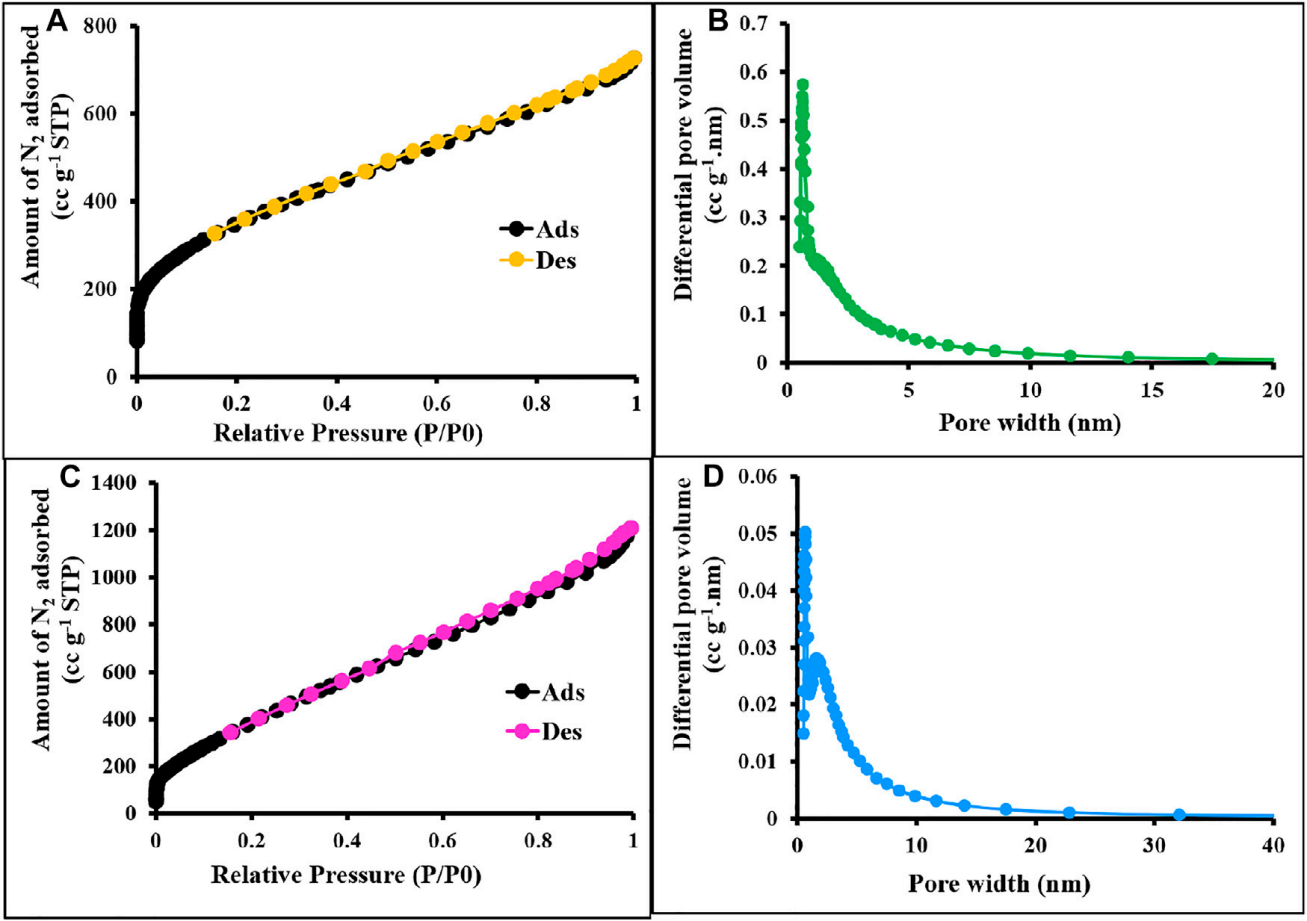
N_2_ sorption–desorption isotherms and PSD of c-2CzPN–KOH **(A,B)** and c-4CzPN–KOH **(C,D)** at 77 K.

The textural properties of the prepared materials were quantified by using nitrogen adsorption/desorption isotherms measured at 77 K (Zhang et al., 2019), and the values are displayed in Table 1. As shown in Figures 12, 13, all the synthesized materials p-2CzPN, p-4CzPN, c-2CzPN–KOH, and c-4CzPN–KOH resulted in a nitrogen gas adsorption isotherm with a sharp gas uptake at a relatively low pressure (P/P0 < 0.001), representing that the resulting materials mostly preserve microporosity. The sharp increase in nitrogen adsorption at a comparatively high pressure (P/P0 > 0.8) could be attributed to nitrogen condensation in void volumes from the space-inefficient polymer packing. Hysteresis was observed upon desorption for all the materials, with a swelling effect or trapping effect due to gas sorption. Usually, the carbazole-based polymer networks show good Brunauer–Emmett–Teller (BET) specific surface area. Here, the synthesized polymer networks p-2CzPN and p-4CzPN showed a specific surface area of 532 and 693 m^2^ g^−1^, respectively (Figures 2A,C). This prepared network polymer showed better values of surface area than those of other reported carbazole-based porous polymers. Micropore surface area calculation (t-plot analysis) showed around more than 50% of the contribution by the micropore area. Meanwhile, the specific surface area of 1,225 and 1,279 m^2^ g^−1^ was obtained for c-2CzPN–KOH and c-4CzPN–KOH, respectively. It was well noted that the specific surface area was increased twofold in the pyrolyzed samples (Figures 3A,C). In addition, the micropore surface area of carbon materials obtained is higher than that of the BET surface area, which could be due to the dominant formation of mesopores over micropores during pyrolysis. As reported by Shereen *et al.*, the structure of carbazole phthalonitrile obtained from single crystal diffraction analysis gave rise to two equivalent phthalonitrile molecules per asymmetric units. The crystal structure also shows that the orientation of the peripheral carbazole unit is twisted about 50° with reverence to the phthalonitrile plane due to steric constraints, which lead to a good surface area and other textural properties (Majeed et al., 2019). Based on the precursor molecule, the prepared carbon materials show the same textural properties.

**TABLE 1:** Textural properties of p-2CzPN, p-4CzPN, c-2CzPN–KOH, and c-4CzPN–KOH.

Material	S_BET_ ^a^ (m^2^ g^−1^)	S_micro_ ^b^ (m^2^ g^−1^)	V_total_ ^c^ (cm^3^ g^−1^)	Pore size^d^ (nm)
p-2CzPN	532	332	0.28	1.2
p-4CzPN	693	386	0.45	1.3
c-2CzPN–KOH	1,225	1,315	1.12	2.6
c-4CzPN–KOH	1,279	2,351	1.81	4.4

a Specific surface area obtained using the Brunauer–Emmett–Teller method.

b Micropore surface area achieved through the *t*-plot method.

c Total pore volume measured at P/P_0_ = 0.9.

d Pore size distribution calculated using the NLDFT method.

Figure 2 represents the sorption isotherms and pore size distribution (PSD) of the prepared materials. The obtained isotherm is type I with no hysteresis upon desorption attributed to the complete physisorption, and the highest nitrogen uptake occurs at P/P0 < 0.03 ascribed to the high population of micropores. The pore size distribution was measured using NLDFT analysis. The pore size measurements exposed the uniform pore size distribution for p-2CzPN and p-4CzPN with a central pore width of 1.2 and 1.3 nm, respectively (Figures 2B,D). In addition, the carbon materials exhibit a pore size distribution of 2.6 and 4.4 nm for c-2CzPN-KOH and c-4CzPN-KOH, respectively (Figures 3B,D). However, both the nitrogen sorption–desorption isotherm and PSD studies demonstrated that all the prepared materials own ordered porous structures with micro- and mesopores. The similitude of micropores of the prepared polymers was possibly obtained through the catenation and predicament of their conjugated backbone. The micro- and mesoporous nature of carbon materials may due to the swelling effect (Perrier et al., 2018).

### Carbon Dioxide Adsorption Measurements

The role of nitrogen species and its enrichment in microporous polymer networks for CO_2_ adsorption and separation is still an uncertain question that may create many discussions on CO_2_–nitrogen interaction and heat of adsorption. For instance, the presence of nitrogenous species such as pyridinium, triazine, nitrile, azo, imide, and carbazole moieties in the porous polymer architectures is beneficial to improve their affinity toward CO_2_ gas. In particular, the selective uptake of CO_2_ over N_2_ may arise from enhanced acidic CO_2_–basic interactions. Therefore, it is worth modifying these specialties at the molecular level into porous organic polymers that can have a solid influence on their CO_2_ gas uptake and separation performances (Lu et al., 2021).

**FIGURE 4:**
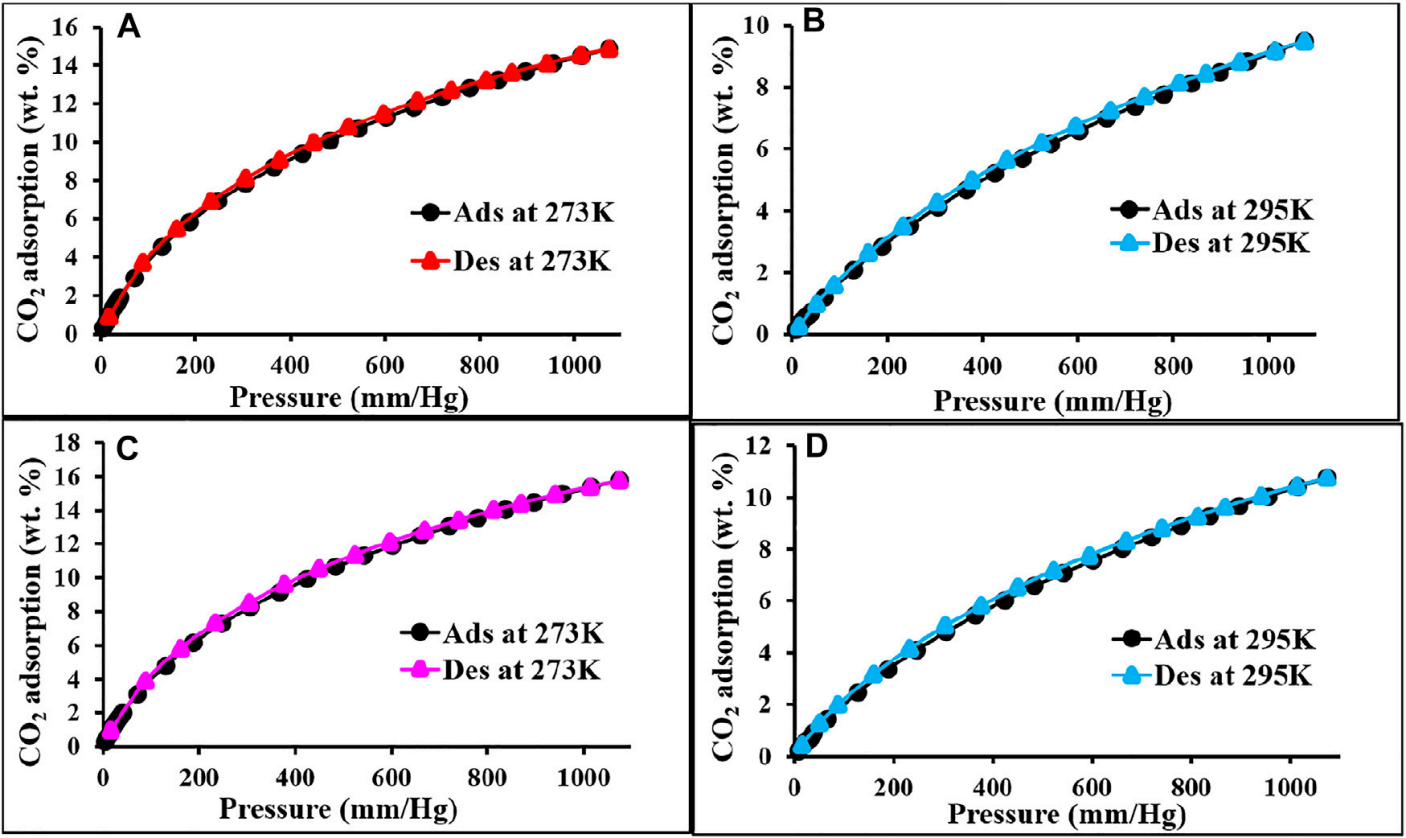
CO_2_ adsorption and desorption isotherm of p-2CzPN **(A,B)** and p-4CzPN **(C,D)** at 273 and 295 K, respectively.

**FIGURE 5:**
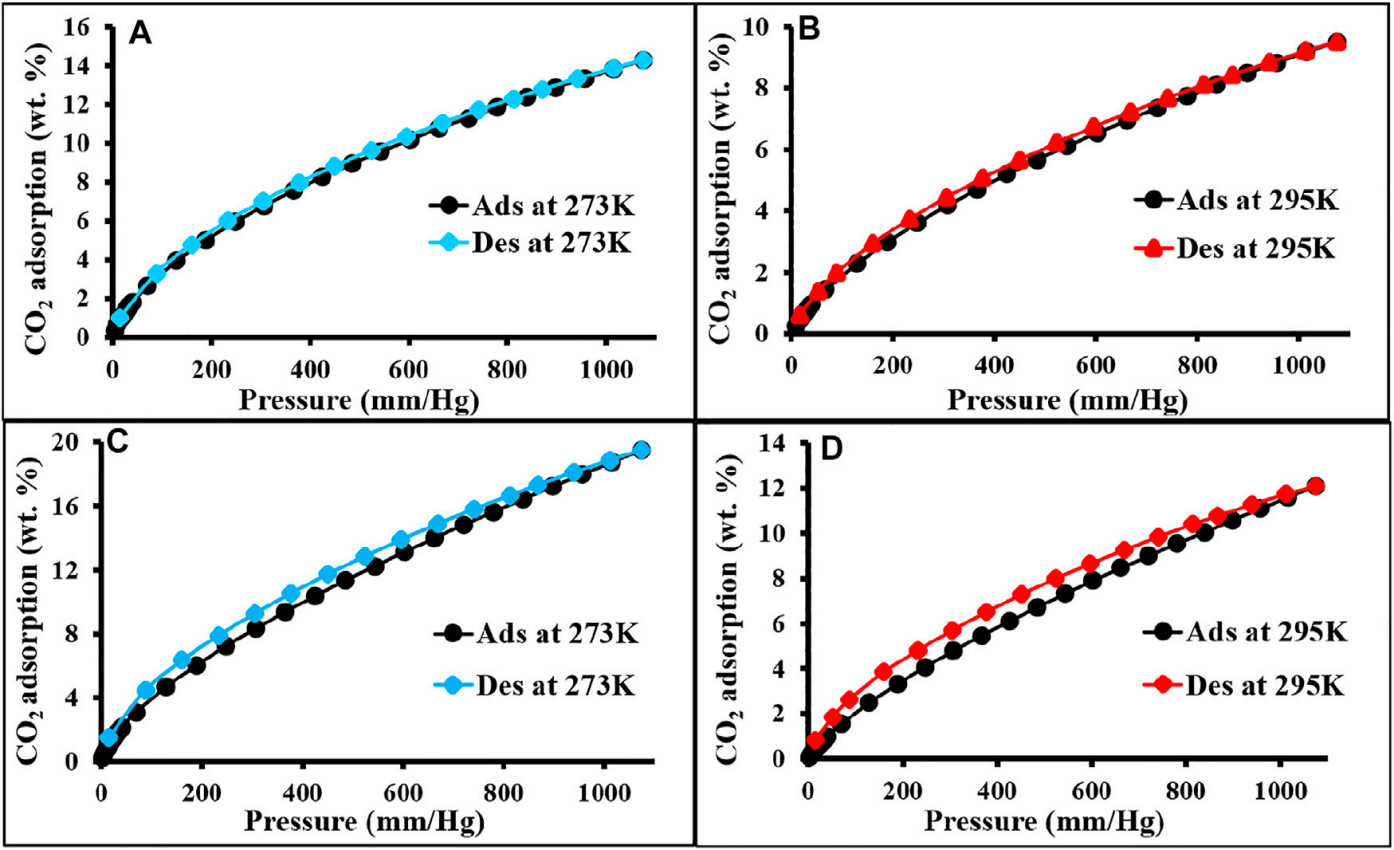
CO_2_ adsorption and desorption isotherm of c-2CzPN–KOH **(A,B)** and c-4CzPN–KOH **(C,D)** at 273 and 295 K, respectively.

The CO_2_ adsorption performance for all the materials was calculated up to 1 bar at 273 and 298 K (Figure 4 and Figure 5). The synthesized polymer networks exhibited the moderate CO_2_ uptake of 14.8 and 15.9 wt% at 273 K for p-2CzPN and p-4CzPN, respectively (Table 2). As shown in Figure 5, the prepared porous carbons show a CO_2_ adsorption capacity of 14.2 and 19.5 wt% at 273 K for c-2CzPN–KOH and c-4CzPN–KOH, respectively. At 295K, c-2CzPN–KOH and c-4CzPN–KOH revealed the maximum CO_2_ uptake capacity of 9.5 wt% and 12.1 wt%, respectively. Comparatively, c-4CzPN–KOH exhibits higher CO_2_ uptake than c-2CzPN–KOH at both 0 and 25°C. The obtained CO_2_ adsorption capacities for c-4CzPN–KOH are higher or comparable to various N-doped carbon sorbents reported in the literature. The detailed comparison of the CO_2_ uptake capacity between the prepared c-4CzPN–KOH and other adsorbents is shown in Table 3.

**TABLE 2:** CO_2_ adsorption capacities of p-2CzPN, p-4CzPN, c-2CzPN–KOH, and c-4CzPN–KOH.

Material	CO_2_ uptake in wt% at 273 K	CO_2_ uptake in wt% at 295 K	Heat of adsorption (kJ mole^−1^)
p-2CzPN	14.8	9.4	33.3
p-4CzPN	15.7	10.7	29.4
c-2CzPN–KOH	14.2	9.5	29.6
c-4CzPN–KOH	19.5	12.1	35.7

**TABLE 3:** Comparison of the CO_2_ uptake and electrochemical performance of the carbon materials reported in the literature.

Material	SA_BET_^a^	CO_2_ uptake^b^ in wt%	Electrolyte	Current density	Specific capacitance (F g^−1^)	Ref
CK-900	1860	30.4	1 M Na_2_SO_4_	0.5 A g^−1^	120	Nazir et al., 2020
LSM-550–2	1941	28.9	6 M KOH	0.5 A g^−1^	325	Li et al., 2021
CX-HMTA4	963	15.5	6 M KOH	1 A g^−1^	161	Wang S. et al., 2021
NPC-2	3,038	20.2	6 M KOH	1 A g^−1^	307	Mishra et al., 2021
N, O-PC-CNTs	2,164	25.1	6 M KOH	0.2 A g^−1^	287	Hao et al., 2020
GCF-0.2	770	21.9	6 M KOH	1 A g^−1^	173	Zhang et al., 2021
ACBS-6	2,335	23.2	3 M KOH	1 A g^−1^	264	Li et al., 2020
NC-850	901	17.1	0.5 H_2_SO_4_	0.5 A g^−1^	185	Meng et al., 2022
DC-2	1968	19.3	6 M KOH	0.5 A g^−1^	222	Bai et al., 2021
c-4CzPN–KOH	1,279	19.5	6 M KOH	1 A g^−1^	451	This work

**FIGURE 6:**
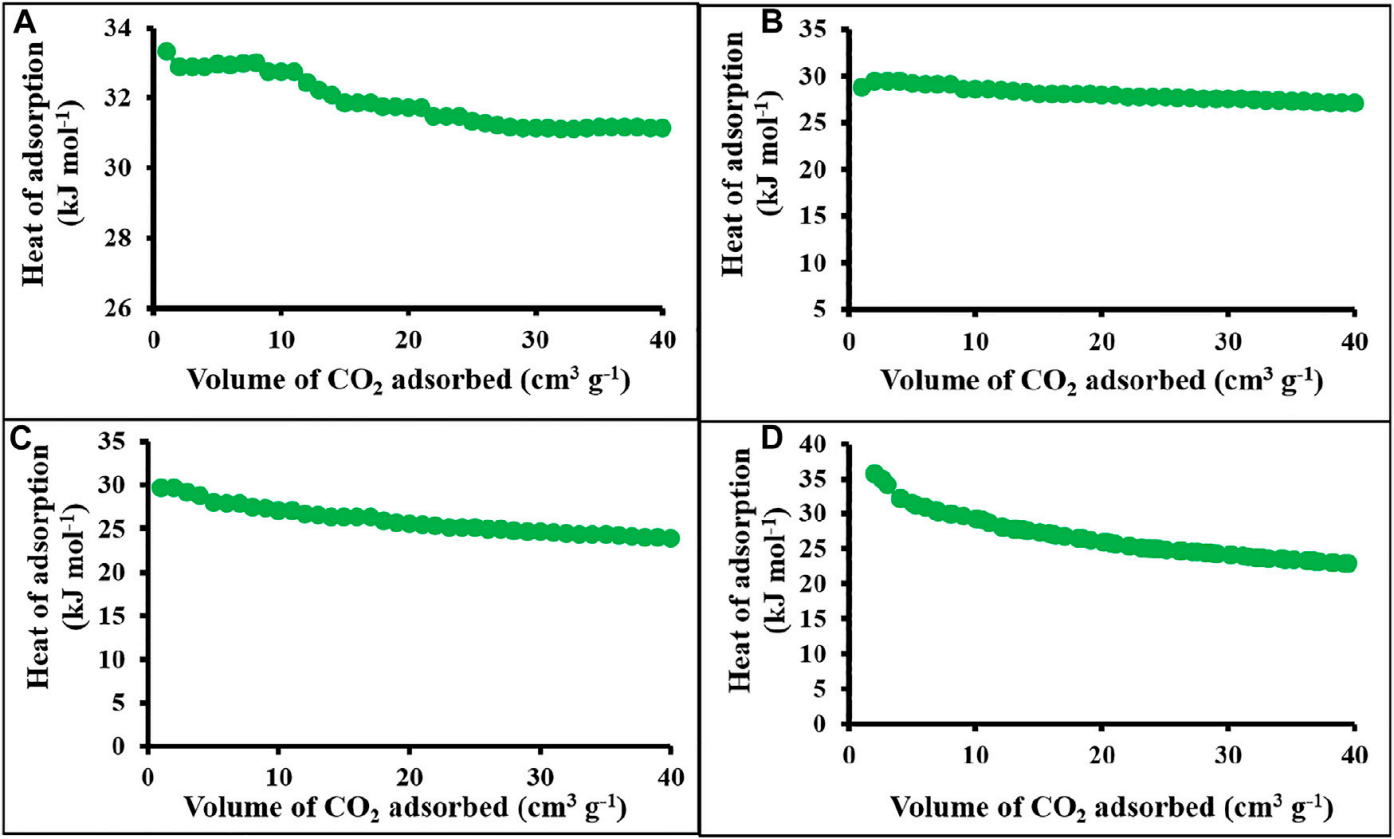
Heat of adsorption plots of **(A)** p-2CzPN, **(B)** p-4CzPN, **(C)** c-2CzPN–KOH, and **(D)** c-4CzPN–KOH.

The calculated heat of adsorption for polymer networks and carbonaceous materials was approximately 29.4–35.7 kJ mole^−1^ (Figure 6). The highest heat of adsorption obtained for c-4CzPN-KOH is 35.7 kJ mole^−1^ (Figure 6D). At initial loading, the host–guest interaction occurs with the most energetically favored sites. The reasonable heat of adsorption obtained is the result of the CO_2_–nitrogen species interaction and the predominant population of sub-nanometer pores that allow the affinity for the attractive proximal surfaces. The higher affinity of carbon dioxide molecules may result from the polar nature of CO_2_, which possesses a quadruple moment that contributes positively toward the CO_2_ selectivity because of the probable interaction between basic nitrogen and acidic CO_2_ molecule (Saha and Kienbaum, 2019).

### CO_2_/N_2_ Selective Adsorption Measurements

In order to find the propensities of the synthesized porous carbons for selective CO_2_ capture, the nitrogen adsorption experiments were performed at 278 K. The CO_2_/N_2_ selectivity of c-2CzPN–KOH and c-4CzPN–KOH carbon materials was measured using the Henry’s law initial slope technique at 0°C (Supplementary Figure S23). The CO_2_/N_2_ selectivity calculated for c-2CzPN–KOH and c-4CzPN–KOH is 30.55 and 66.56, respectively. The ample uniform pores and high affinity functionalities are significant factors for the selective adsorption of CO_2_. Here, the attained c-4CzPN–KOH shows twice the CO_2_ selectivity performance than c-2CzPN–KOH. This could be due to the role of larger pore size. Notably, c-4CzPN–KOH exhibits a larger pore size than c-2CzPN–KOH. Interestingly, the precursors (p-2CzPN and p-4CzPN) used for the synthesis of carbonaceous materials contain the same functionalities. However, during the pyrolysis process, the formation of enriched active nitrogen species is found well in c-4CzPN–KOH. The enriched proportion of nitrogen species and larger pore size distributions can afford the absolute space of the CO_2_ molecules inside the pores. Overall, the CO_2_ uptake studies demonstrated that there was a clear relation between the BET surface area and CO_2_ uptake. Additional factors such as sub nanopore dimensions and micropore surface area facilitate the improved CO_2_ adsorption. Moreover, the CO_2_ adsorption isotherms of the materials were fully reversible and no trapping effect was found. A linear correlation between the CO_2_ adsorption capacity and micropore volume was observed, which suggested that the population of the micropore should be important for the CO_2_ adsorption. It is well known that the presence of the doped nitrogen could provide attractive sites for CO_2_ molecules, and higher nitrogen content means more CO_2_ adsorption sites. Here, the prepared c-4CzPN–KOH exhibited enriched nitrogen species especially pyrrolic and pyridinic N1s during carbonization, which may help the material for improved CO_2_ uptake (Petrovic et al., 2021).

**FIGURE 7:**
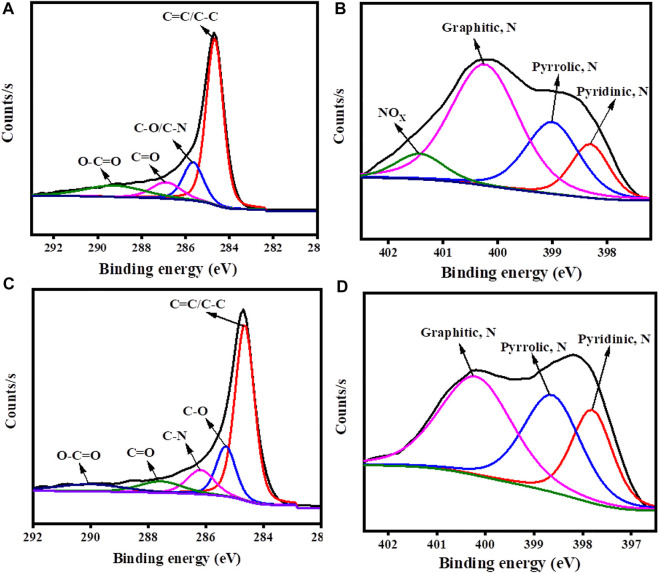
C1s, N1s deconvoluted XPS spectrum of **(A,B)** c-2CzPN–KOH and **(C,D)** c-4CzPN–KOH.

In order to find the chemical state of the atoms such as C 1s and N 1s, the deconvoluted XPS spectrum was performed for carbon materials. The XPS C 1s core level peak of c-2CzPN–KOH could be deconvoluted into four peaks at 284.6 (C=C, *sp* ^2^), 285.6 (C-C, *sp* ^3^), 286.8 (C=O), and 288.1 eV (O-C=O) (Figure 7A). Similarly, the XPS C 1s core level peak of c-4CzPN–KOH can be deconvoluted into five peaks (Figure 7C) at 284.6 (C=C, *sp* ^2^), 285.3 (C-C, *sp* ^3^), 286.1 (C=O), 287.5 (C=N), and 288.1 eV (O-C=O). Subsequently, the N 1s spectra of c-2CzPN–KOH could be split into four peaks, corresponding to pyridinic N (398.3 eV), pyrrolic N (399.1 eV), graphitic N (400.2 eV), and NOx (401.4 eV) (Figure 7B). The N 1s peaks of c-4CzPN–KOH are shown in Figure 7D. It had three nitrogen functional groups, centered at 397.8, 398.6, and 400.2 eV, which were also assigned to pyridine-N, pyrrolic-N, and graphitic-N, respectively. Here, pyridine-N means a nitrogen atom bonded to 2 C atoms in a hexagonal ring, and the peak recorded at 400 eV refers to graphitic nitrogen that is sited inside the carbon structure. The peak obtained at the highest binding energy was consistent with N-oxides of pyridine-N (NOx) (Ayiania et al., 2020). Based on available reports, pyridine-N and pyrrolic-N have been shown to have a positive effect in enhancing capacitive performance *via* pseudocapacitance due to split electron pair configuration effects. Moreover, the introduction of quaternary N could also facilitate electron transfer and improve the conductivity of carbonaceous materials. In the present work, the proportions of pyrrolic- and pyridine-N obtained for c-4CzPN–KOH are higher than those of c-2CzPN–KOH, which can help the material achieve better capacitance performance.

The prepared monomer was stated for organic electronics (Treeweranuwat et al., 2020). Therefore, the network polymers prepared using the monomer can be highly conductive in nature. In order to find the preliminary conducting property of the polymers, ultraviolet photoelectron spectroscopy (UPS) studies were achieved to find the molecular orbital energies between 2 and 4 eV (valence band region) (Supplementary Figure S24). The regions between 2 and 4 eV were considered highly occupied molecular orbital (HOMO) states, and all materials show a valence band around 2.67 eV. This could be due to the existence of robust π - π * conjugation in the polymer backbone (Wex and Kaafarani, 2017). In case of carbonaceous materials, c-4CzPN–KOH exhibits a clear peak in the valence band region than c-2CzPN–KOH (Supplementary Figure S24B). This could be due to the presence of enriched N species in the c-4CzPN–KOH carbon network.

### Capacitive Performance of p-2CzPN and p-4CzPN Polymers

**FIGURE 8:**
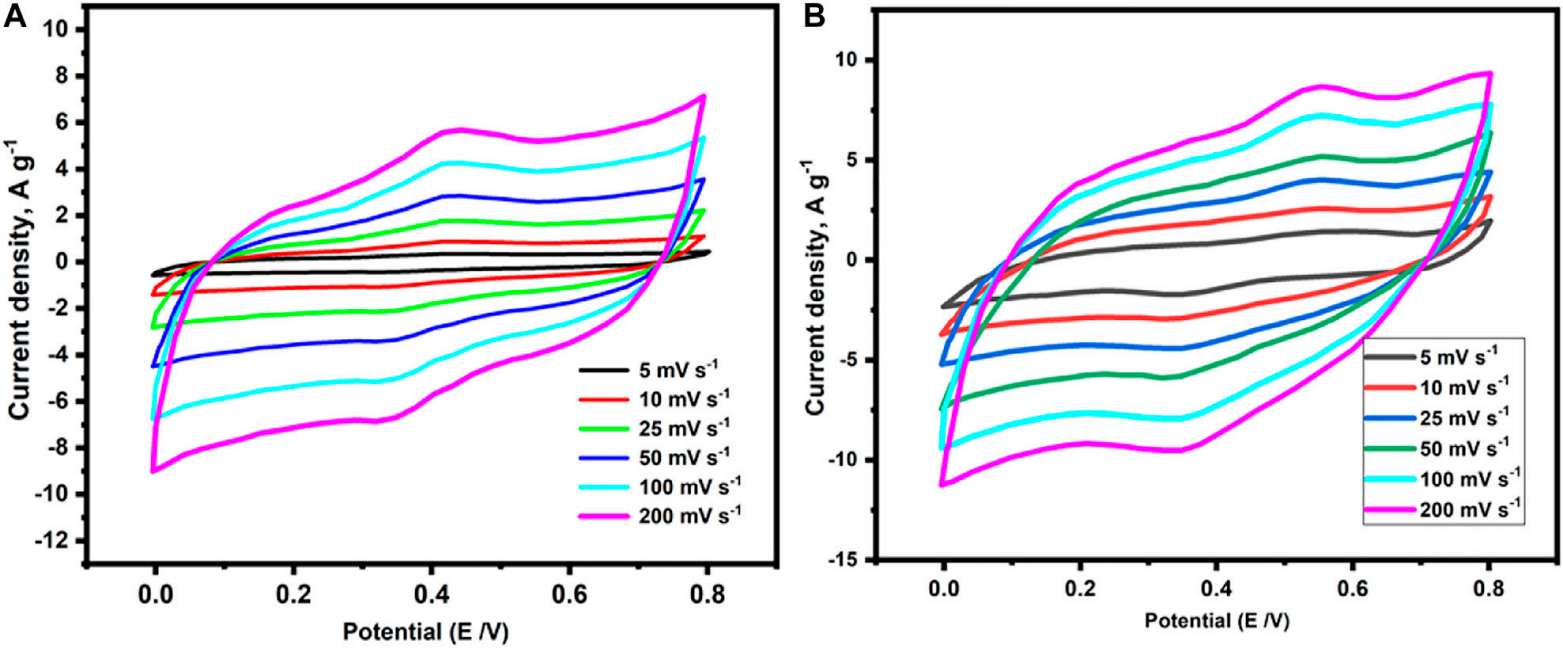
Cyclic volumetric curves of **(A)** p-2CzPN and **(B)** p-4CzPN polymers attained at different scan rates.

**FIGURE 9:**
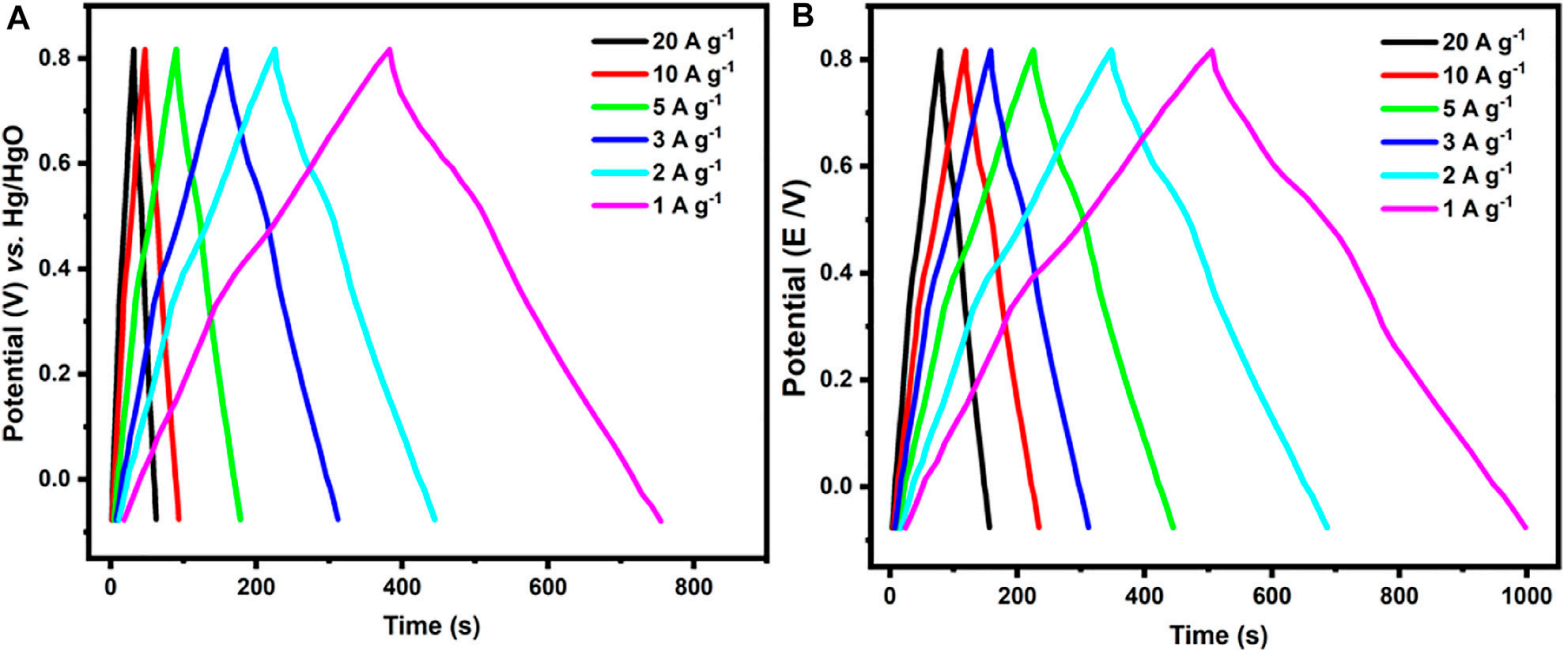
GCD curves of **(A)** p-2CzPN and **(B)** p-4CzPN polymers tested at various current densities.

In an electrical double layer capacitor (EDLC)-based supercapacitor, large surface area and well-ordered pore size electrodes are the key parameters for remarkable electrode efficiency. Furthermore, porous organic polymers (POP) have recently attracted great attention as electrode materials owing to their capacity to integrate redox-active moieties in their networks (Zhang et al., 2015). Accordingly, the investigated conjugated POPs (p-2CzPN and p-4CzPN) include a redox-active carbazole moiety and a rich nitrogen content in its skeleton, as well as a high specific surface area and consistent pore size. These POPs (p-2CzPN and p-4CzPN) were tested as supercapacitor electrodes, and their electrochemical properties were determined by utilizing cyclic voltammetry (CV) (Figure 8) and galvanostatic charge–discharge (GCD) (Figure 9) experiments in a three-electrode system with 6.0 M KOH electrolyte (Mohamed et al., 2022).

The CV of p-2CzPN and p-4CzPN was obtained in the potential range of 0–0.8 V at various sweep rates (5, 10, 25, 50, 100, and 200 mV s^−1^) with Hg/HgO electrode as a reference electrode. As depicted in Figure 8, rectangle-like CV plots were observed, confirming that the capacitive response of the investigated polymers arises from EDLC. According to previous studies, EDLC is caused primarily by the creation of electrostatic double layers at the electrode/electrolyte interface. Consequently, the EDLC behavior of the investigated polymers (p-2CzPN and p-4CzPN) may be ascribed to the development of electrostatic double layers among the electrolyte and polymeric film. Furthermore, the existence of N atoms in the polymer’s framework increases the interlayer distances among the polymeric layers, resulting in greater ion diffusion and electron transport into the polymer’s films. These characteristics are predicted to result in a supercapacitor electrode with excellent capacitance performance while using these polymers. The rectangle-like form of the CV curves was maintained at all sweep rates, even at the maximum one (200 mV s^−1^), demonstrating good charge transmission through the polymeric electrodes. Furthermore, our investigated polymer’s CV plots included a tiny hump, indicating a combination of small pseudocapacitance and significant EDLC. These humps were produced by Faradic redox currents, which might have been caused by the existence of redox-active carbazole units in the polymer’s backbone.

Moreover, GCD experiments were carried out to validate the capacitive characteristics of the investigated polymers at various current densities (1, 2, 3, 5, 10, and 20 A g^−1^), as shown in Figure 9 (Jing et al., 2021). In harmony with the CV results, the polymeric electrodes displayed triangular charge–discharge plots with a small bend, demonstrating a combination of EDLC and pseudocapacitive performance induced by Faradic electrochemical redox processes. The specific capacitances derived from the GCD curves for p-2CzPN and p-4CzPN electrodes at a current density of 1 A g^−1^ were 272 g^−1^ and 319 F g^−1^, respectively. By increasing the current density up to 20 A g^−1^, the capacitance of the p-2CzPN and p-4CzPN electrodes decreased to 110 and 192 F g^−1^, respectively.

**FIGURE 10:**
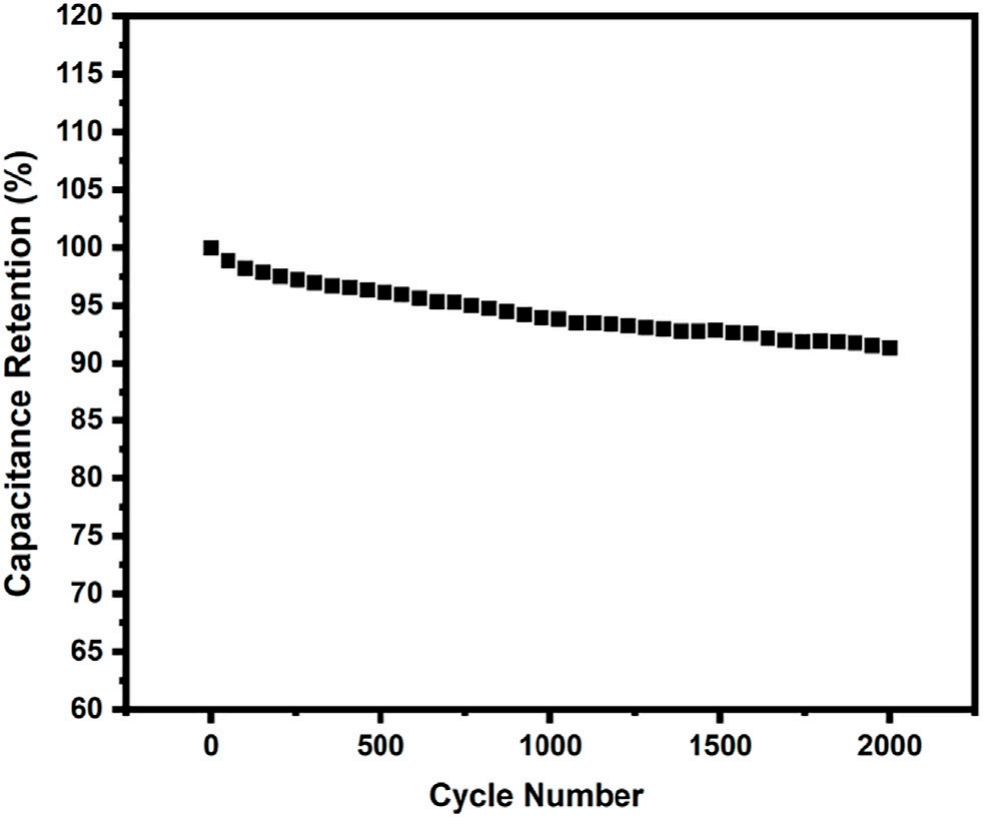
Cyclic stability of p-4CzPN polymer at a current density of 2 A g^−1^ (retained about 91% after 2000 cycles).

The long-term cycle stability of the p-2CzPN and p-4CzPN electrodes was measured by utilizing 2000 charge/discharge cycles at a steady current density of 2 A g^−1^. The p-4CzPN electrode demonstrated excellent stability of about 91% capacitance retention, as illustrated in Figure 10.

### Capacitive Performance of c-2CzPN–KOH and c-4CzPN–KOH Carbon Materials

**FIGURE 11:**
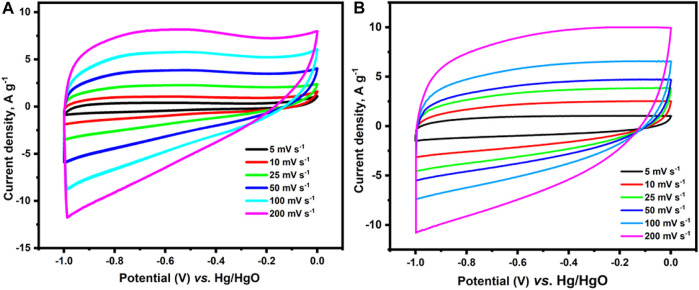
Cyclic voltametric figures of **(A)** c-2CzPN–KOH and **(B)** c-4CzPN–KOH attained at various sweep rates.

To broaden the application of p-2CzPN- and p-4CzPN-derived carbon materials, we explored it as electrode materials for supercapacitor application. The impact of the activation base used in the carbonization procedure on the super-capacitive behavior of the explored compounds was explored depending on the construction of the carbon compounds with high nitrogen content, numerous micropores, and large surface area (Gao et al., 2021). CV scans of c-2CzPN–KOH and c-4CzPN–KOH carbon materials were performed in 6 M KOH in a three-electrode cell design at a sweep rate of 10 mV s^−1^. All the carbon material electrodes demonstrated a quasi-rectangular shape together with a few undefined features, demonstrating that the specific capacitance was improved from combined influences of EDLC and pseudocapacitance (Figure 11).

**FIGURE 12:**
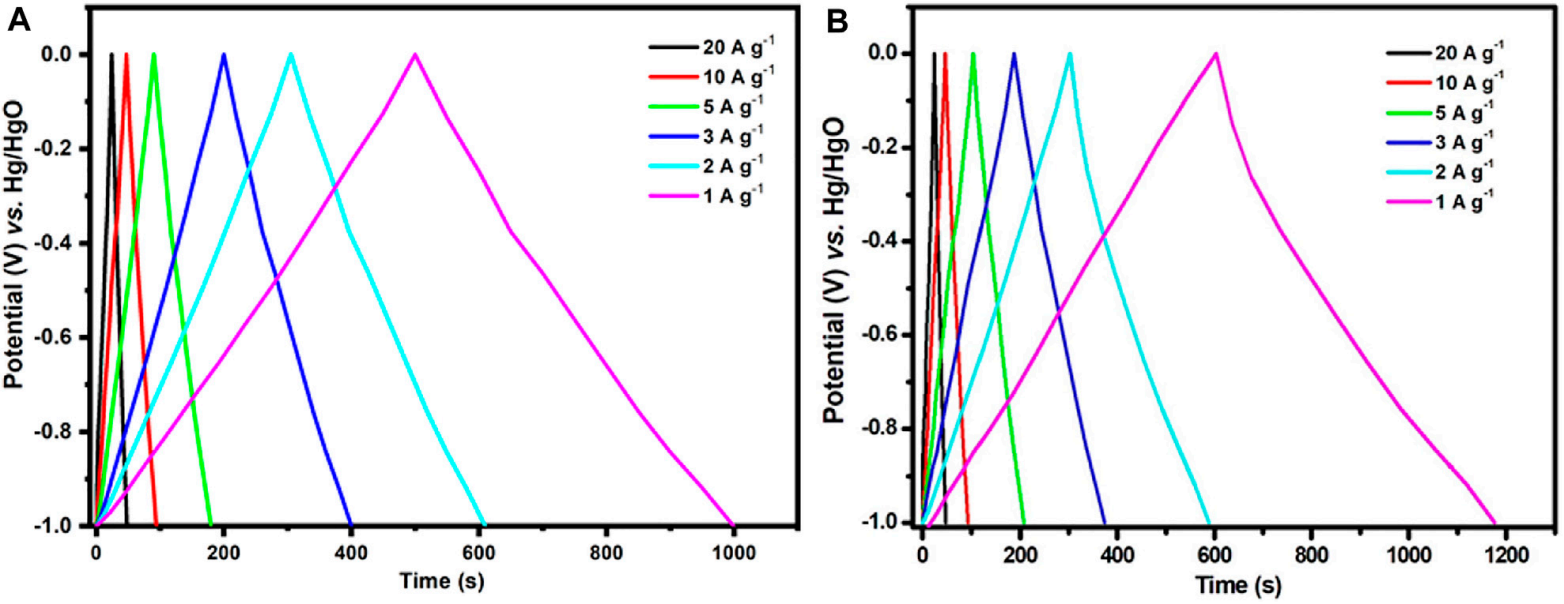
GCD curves of **(A)** c-2CzPN–KOH and **(B)** c-4CzPN–KOH tested at various current densities.

The CV plots of c-4CzPN–KOH maintained the rectangular shape with variations in the sweep rates (5–200 mV s^−1^), indicating excellent capacitive performance and excellent reversibility, as shown in Figure 11. Despite minor variations at higher scan speeds, the rectangle-like form was maintained, suggesting high-rate capabilities and rapid ion transport. Moreover, the porous structure of these compounds enables dissolved ions to interact with the electrode surfaces, boosting electrochemical capacitance. c-4CzPN–KOH demonstrated the largest area under the CV plots when compared to c-2CzPN–KOH carbon materials, implying the greatest electrochemical capacitance owing to its largest surface area and nitrogen content. Furthermore, the specific capacitances were determined using galvanostatic charge/discharge (GCD) experiments in 6.0 M KOH utilizing a three-electrode cell setup (Figure 12). All porous carbons exhibited a symmetric triangular shape. According to the recorded results, c-4CzPN–KOH demonstrated a prolonged discharge time at 1.0 A g^−1^, compared to c-2CzPN–KOH, which is consistent with the CV findings. The specific capacitance was calculated utilizing the equation C = I/(dV/dt) in charge–discharge tests, where I is the discharging current per mass unit delivered to the electrode and dV/dt is the slope during the voltage decrease in charge–discharge studies. The calculated specific capacitance of c-2CzPN–KOH is 336 F g^−1^ at 1.0 A g^−1^, and c-4CzPN–KOH exhibits 451 F g^−1^ at 1.0 A g^−1^.

**FIGURE 13:**
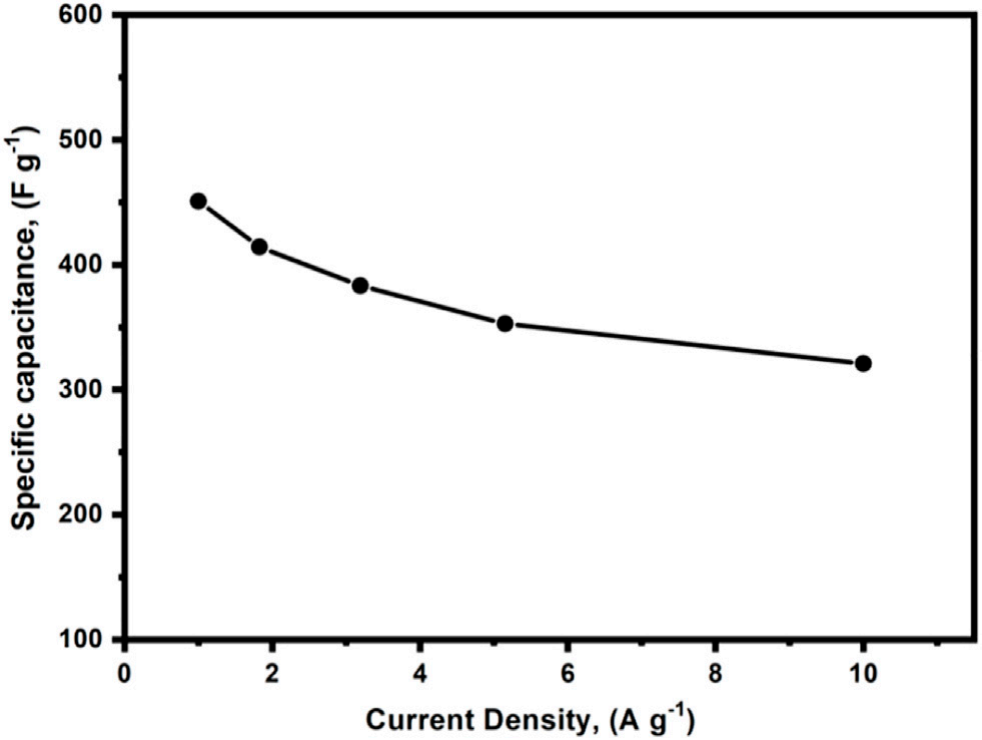
Specific capacitance profiles of c-4CzPN–KOH recorded at different current densities.

Furthermore, the GCD diagram retains a similar linear shape at a high current (10 A g^−1^) including low IR drop, indicating good high-rate performance (Figure 12). As the current density was raised from 1.0 A g^−1^–10 A g^−1^, the capacitance of c-4CzPN–KOH decreased from 451 F g^−1^–321 F g^−1^, as shown in Figure 13. c-4CzPN–KOH maintained a high capacitance with increasing the current density while the decrease in capacitance was believed to be due to a shortage of time for fast ion diffusion and electron transport, which resulted in an increase in inner resistance and a decrease in capacitance. Moreover, the porous structures of the investigated carbon materials are beneficial for supercapacitors as they facilitate ion transport.

**FIGURE 14:**
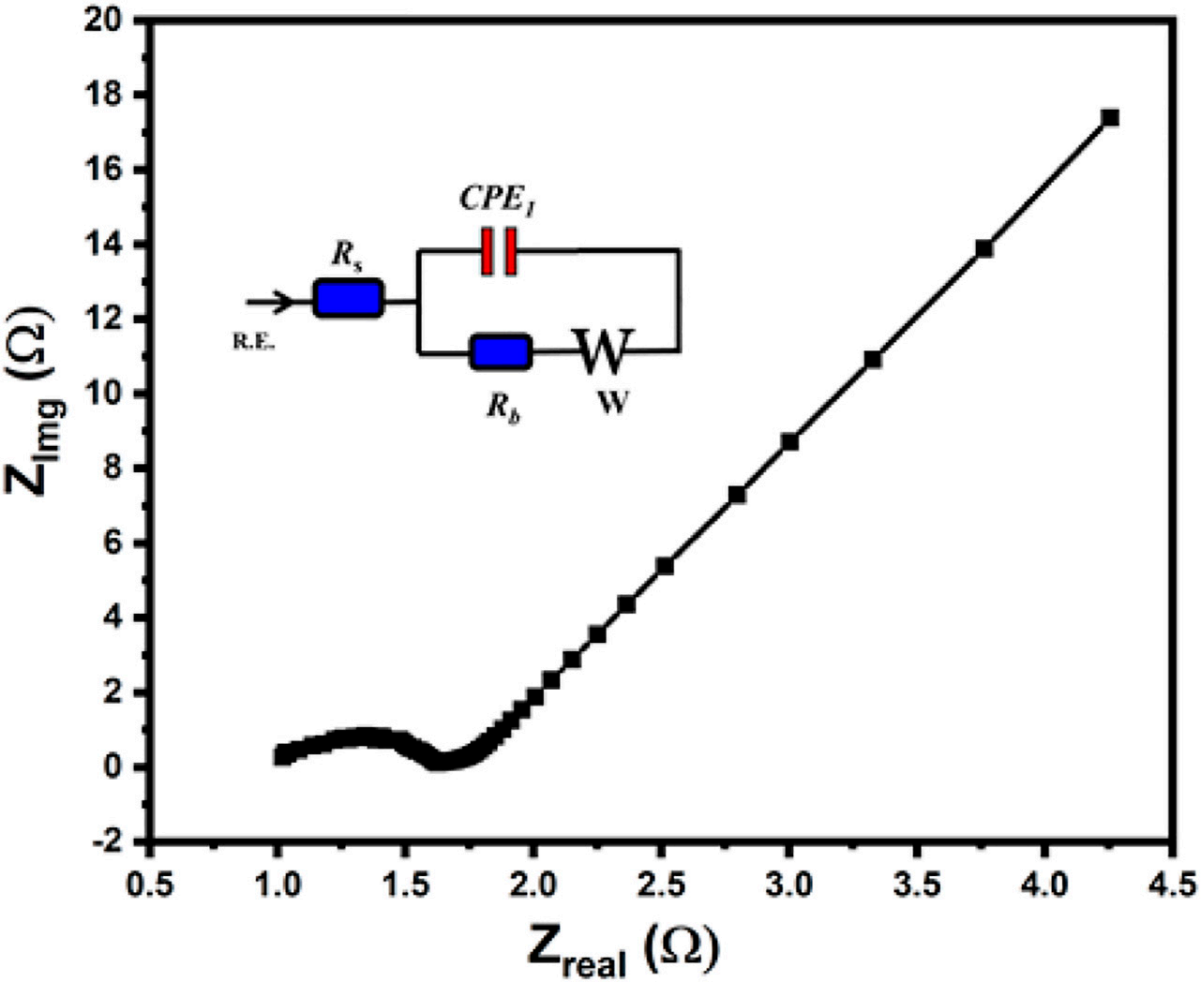
Nyquist plot for c-4CzPN–KOH recorded in 6.0 M KOH. The circuit used for fitting the EIS results is shown in the inset.

To evaluate the resistive properties of the examined carbon materials, EIS measurements were also performed in 6.0 M KOH in a three-electrode system (Ramirez et al., 2020). The Nyquist plot of c-4CzPN–KOH is displayed in Figure 14. In the low-frequency region, the vertical line implies good ion diffusion and suggests extreme capacitive performance. Moreover, in the high-frequency region, the electrodes acted like pure resistors, which is typical of non-metallic carbons demonstrating strong pore conductivity for the electrolyte ions. The figure showed little variations in the middle range frequencies, indicating low conductivity induced by microporosity and pseudocapacitance, which is consistent with the IR declines reported by GCD results. As a result, we infer that those microporous carbons may considerably increase electrochemical capacitance while impairing ion transport, resulting in charge-transfer resistance. Moreover, cycling stability is also a major challenge for supercapacitors, particularly when pseudocapacitance is present.

**FIGURE 15:**
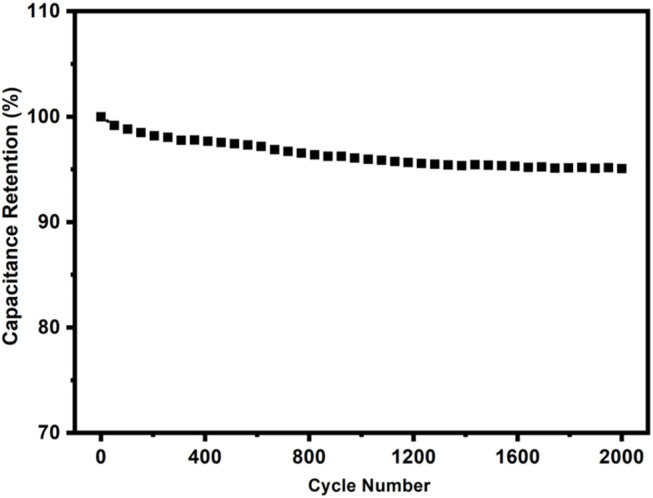
Cyclic stability of c-4CzPN–KOH at a current density of 2 A g^−1^ (retained about 95.9% after 2000 cycles).

The cyclic stability of c-4CzPN–KOH was carried out at a current density of 2.0 A g^−1^ (Figure 15) to examine its electrochemical stability. After 2000 cycles, the capacitance had decreased only 4.1% of the initial value, demonstrating the significant cycle stability of c-4CzPN–KOH carbon material, making it a suitable supercapacitor electrode material.

The aforementioned table describes the CO_2_ gas adsorption and electrochemical performance of different carbonaceous materials reported in recent literature. If we look at Table 3, except for three materials, NC-850, GCF-0.2, and CX-HMNO4, the rest of the materials show higher specific surface area than c-4CzPN–KOH. Meanwhile, all the aforementioned materials show a uniform pore size between 0.8 and 2 nm, except for N, O-PC-CNT, which shows a pore size of 2–4 nm. Moreover, c-4CzPN–KOH reveals a pore size of 4.4 nm, which is comparatively larger than that of all other materials. According to the literature, a porous material with high surface areas provides more active sites for the adsorption/desorption process and ion/electron transport in electrochemical energy storage applications. In case of carbon as an electrode material, however, a carbon with a large surface area, other factors play a crucial role in assessing the efficiency of the material. For instance, in some cases, the micropores cannot sustain the relatively excessive loading of electrolyte species, and the mesopores may adapt well to the process. This can be very favorable for the internal stress and energy storage degradation with constant charge/discharge cycles (Young et al., 2018). Furthermore, the existence of nitrogen species, especially pyridinic-N and pyrrolic-N, was beneficial for high energy storage performance of the material *via* the pseudocapacitance approach. In summary, the synthesized carbonaceous material has a lower specific surface area than the reported materials, but it exhibits a larger pore size that can accommodate maximum ion storage capacity with the additional help of enriched nitrogen species (pyridine-N and pyrrolic-N) present in the pore walls.

## Conclusion

In conclusion, nitrogen-enriched KOH-activated porous carbons with a high surface area were substantially prepared using a direct pyrolysis approach. The BET surface area of the best performed carbon c-4CzPN–KOH prepared by carbonization of the p-4CzPN network polymer reaches 1,279 m^2^ g^−1^. The obtained c-4CzPN–KOH exhibits high surface area, uniform porosity, and shows an excellent CO_2_ capture performance of 19.5 wt% at 273 K. Furthermore, c-4CzPN–KOH was utilized for electrochemical measurements, and it revealed a high capacitance of up to 451 F g^−1^ in 6.0 M KOH aqueous electrolytes due to the synergetic effect of the ability to exhibit double layer and pseudocapacitance. The reported carbon also exhibits excellent charge/discharge cycle stability and retains 95.9% capacity after 2000 cycles. Even though the reported protocols and precursors are simple and known, the opportunity to create carbon materials with a high and customizable specific surface area with uniform pores is still open. Thus, the current work successfully demonstrates the synthesis and utilization of activated carbon from porous network polymers for CO_2_ uptake and the competent carbon electrode for supercapacitors and energy storage applications.

## Data Availability

The raw data supporting the conclusion of this article will be made available by the authors, without undue reservation.
