# Supplementary material for: Development of Uniform Porous Carbons From Polycarbazole Phthalonitriles as Durable CO2 Adsorbent and Supercapacitor Electrodes
Source: Front Chem. 2022 Apr 25;10:879815. doi: 10.3389/fchem.2022.879815 (PMC9081769; doi:10.3389/fchem.2022.879815)
Supplement: Supplementary file 1 [file DataSheet1.docx]

Supporting Information

**Development of uniform porous carbons from polycarbazole phthalonitriles as durable CO_2_ adsorbent and supercapacitor electrodes**

Ghadeer Thani Alenzi, Narendran Rajendran, Ahmed Abdel Nazeer and Saad Makhseed^*^

Department of Chemistry, Faculty of science, Kuwait University, Safat, 13060, Kuwait

* saad.makhseed@ku.edu.kw; Tel: +965 24985538

**Contents**

[**Section A. Synthesis Procedure 2**](#_Toc98428792)

[**Section B. FT-IR Spectral profiles 5**](#_Toc98428793)

[**Section C. Raman analysis 7**](#_Toc98428794)

[**Section D. NMR Profile 8**](#_Toc98428795)

[**Section E. Mass analysis of monomers 15**](#_Toc98428796)

[**Section F. HPLC Profile 17**](#_Toc98428797)

[**Section G. DSC analysis of monomers 18**](#_Toc98428798)

[**Section H. TGA Profiles 19**](#_Toc98428799)

[**Section I. PXRD Profiles 20**](#_Toc98428800)

[**Section J. Particle size analysis 22**](#_Toc98428801)

[**Section K. XPS Profile 23**](#_Toc98428802)

[**Section L. SEM-EDX analysis of the carbon materials 25**](#_Toc98428803)

[**Section M. CO_2_/N_2_ Selectivity studies 27**](#_Toc98428804)

[**References 29**](#_Toc98428805)

# Section A. Synthesis Procedure

**Synthesis of 2CzPN**

4,5-Dichlorophthalonitrile (1g, 5.7mol) and carbazole (2.54g, 15.2mol) was taken in 100mL round bottom flask organized with nitrogen atmosphere and dissolved in 25mL of Dry DMF. To the reaction mixture, Cesium fluoride (7.71g, 50.7mol) was added quickly and stir vigorously at 90°C for 24 hr. The completion of the reaction was confirmed by TLC. After the reaction was completed, the reaction mixture was cooled and transferred to cold water. Filter the pale-yellow precipitate and the excess carbazole was removed by reflux with methanol. Yellow powder; Yield 80%, m.p >300°C; IR/cm^-1^ (KBr): 3045 (aromatic =C-H, str), 2234 (C-N str, nitrile), 1449 (aromatic C=C- str); ^1^H-NMR (DMSO-d6, 600MHz, δppm): 8.83 (s, 1H), 7.92 (t, *J*=1.2 Hz,7.2 Hz, 2H), 7.30 (t, *J* =7.2 Hz, 1.2 Hz, 2H), 7.11-7.06 (m, 4H); ^13^C-NMR (DMSO-d6, 150MHz, δppm): 138.49, 137.96, 136.38, 125.81, 123.23, 120.93, 120.19, 115.32, 115.01, 109.81; HR-Ms calculated ( for C_32_H_18_N_4_): 458.1526, obtained: 458.1526; Purity: 99.44% (HPLC).

**Synthesis of 4CzPN**

For synthesis of 4CzPN, Tetrafluoroterephthalonitrile (1g, 5mol) and carbazole (4.17g, 25mol) was dissolved in dry DMF and add CsF (7.6g, 50mol) to the reaction mixture. The reaction was heated to 90°C for 48 hr at N_2_ atmosphere. The reaction mixture was cooled and transferred to cold water. The orange red precipitate obtained was purified by reflux with methanol. Yellow powder; Yield 80%, m.p >300°C; IR/cm^-1^ (KBr): 3076 (aromatic =C-H, str), 2234 (C-N str, nitrile), 1458 (aromatic C=C- str), 1H-NMR (DMSO-d6, 600MHz, δppm): 7.99 (d, *J*=7.8Hz, 1H), 7.96 (d, *J*=6Hz, 1H), 7.30-7.28 (m, 1H), 7.19-7.16 (m, 1H), HR-Ms calculated (for C_56_H_32_N_6_): 788.2684, obtained: 788.2683; Purity: >99.99% (HPLC).

# Section B. FT-IR Spectral profiles


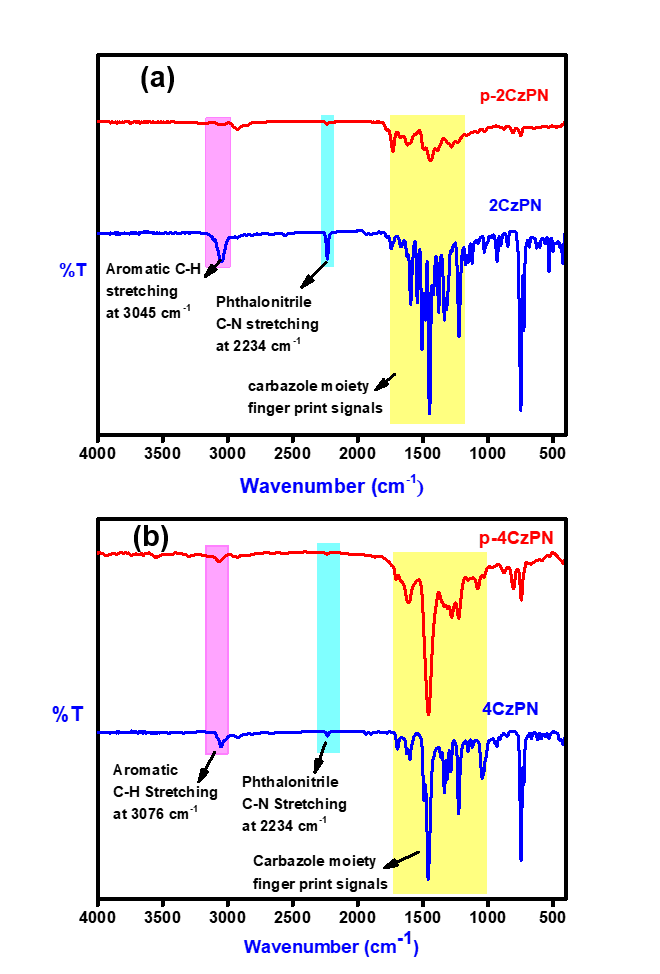


**Fig. S1. FTIR spectrum of (a) 2CzPN, p-2CzPN and (b) 4CzPN, p-4CzPN by KBr pellet method**


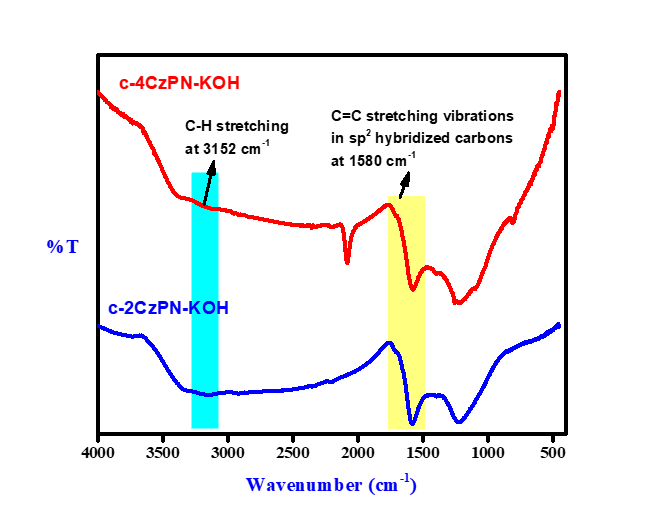


**Fig. S2. FTIR spectrum of c-2CzPN-KOH and c-4CzPN-KOH by KBr pellet method ^[1]^**

# Section C. Raman analysis


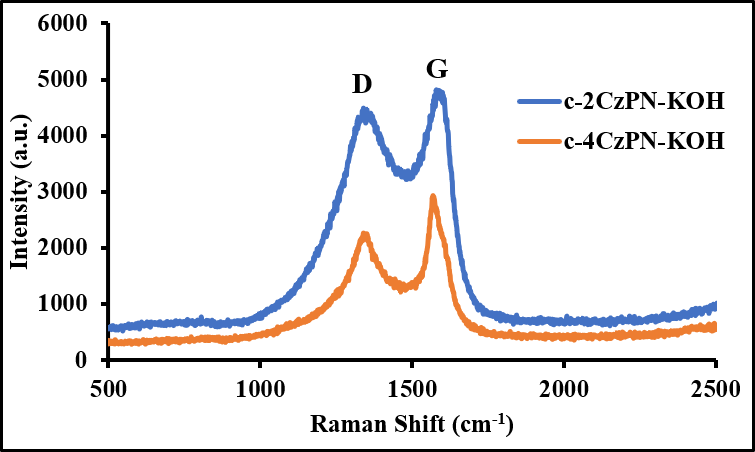


**Fig. S3. Raman spectrum of the carbon materials ^[2]^**

# Section D. NMR Profile

**
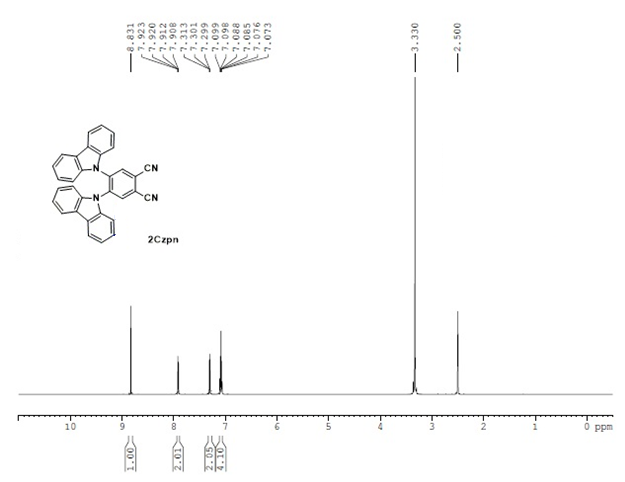
**

**Fig. S4. ^1^H-NMR of the monomer 2CzPN**

**
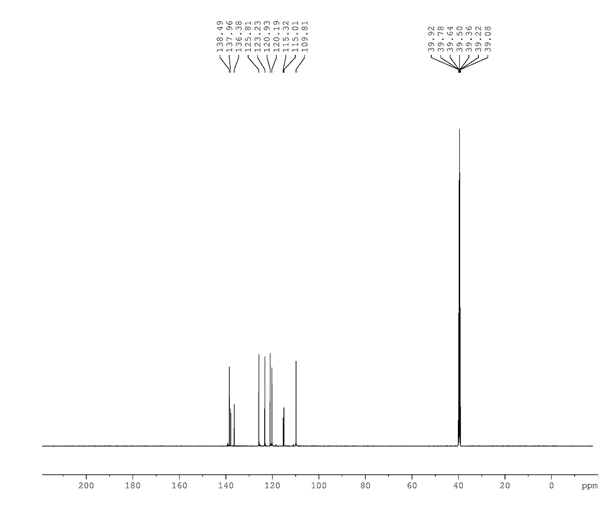
Fig. S5. ^13^ C- Decoupled NMR spectrum of 2CzPN**

**
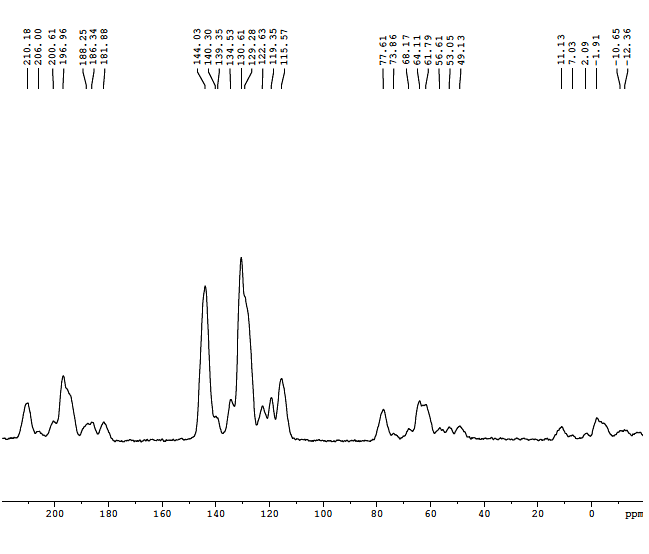
**

**Fig. S6. Solid state ^13^C CP-MAS-spectrum of 2CzPN**

**
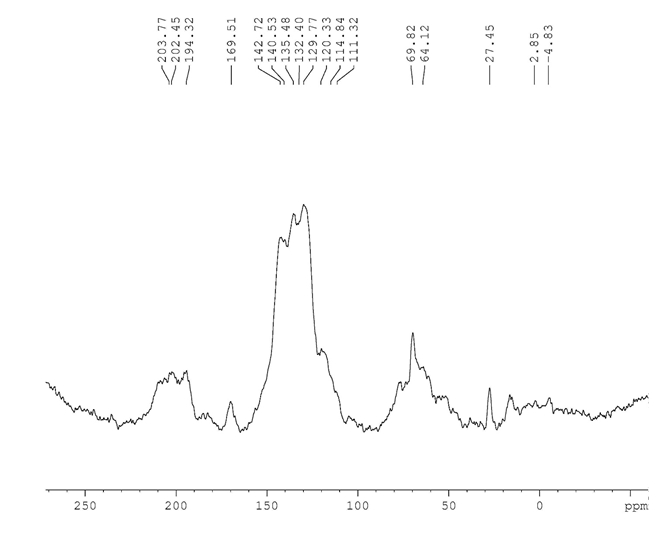
**

**Fig. S7. Solid state ^13^C CP-MAS-spectrum of p-2CzPN**


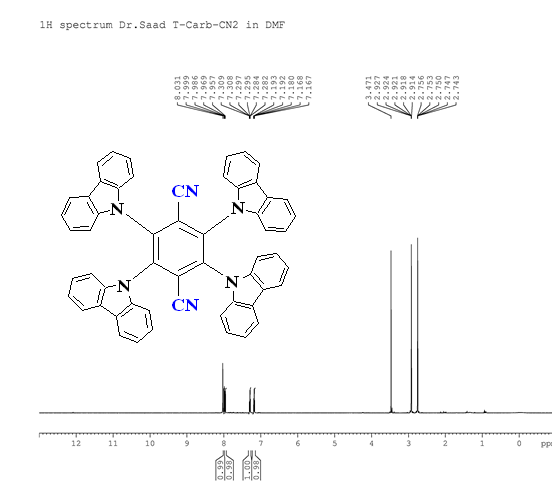


**Fig. S8. ^1^H-NMR of the monomer 4CzPN**

**
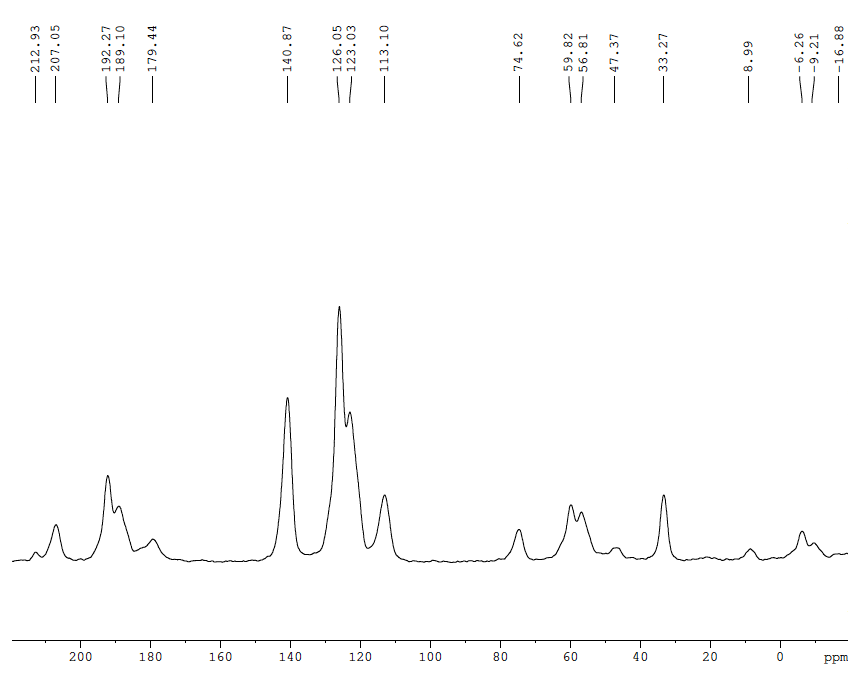
**

**Fig. S9. Solid state ^13^C CP-MAS-spectrum of 4CzPN**

**
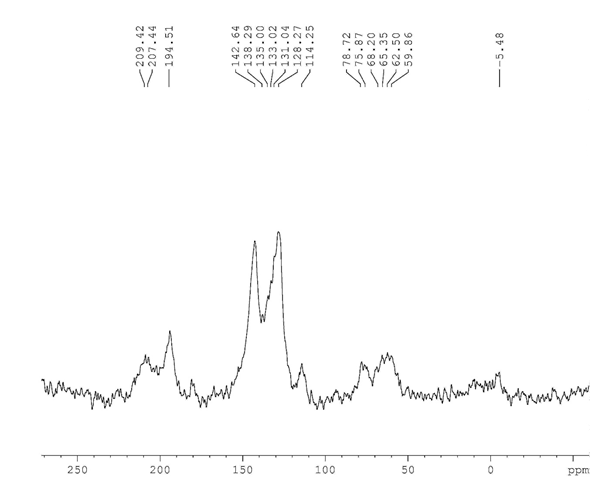
**

**Fig. S10. Solid state ^13^C CP-MAS-spectrum of p-4CzPN**

# Section E. Mass analysis of monomers


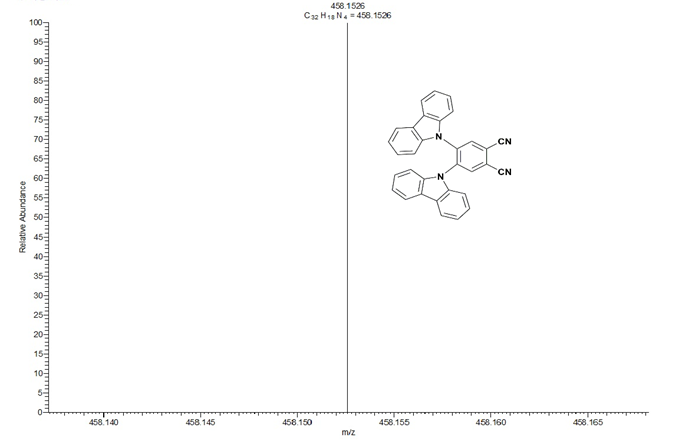


**Fig. S11. HR-MS analysis of 2CzPN**


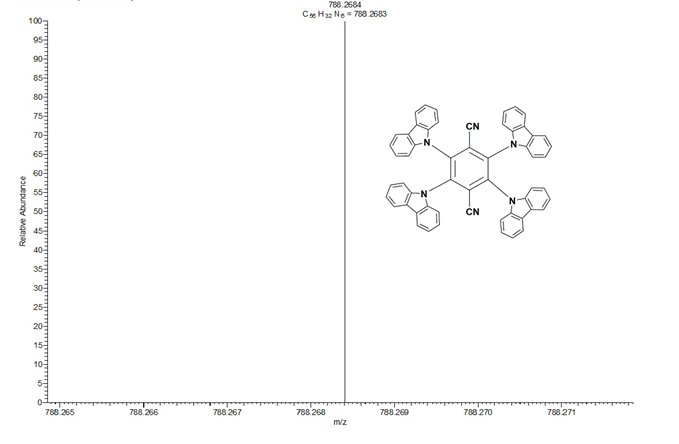


**Fig. S12. HR-MS analysis of 4CzPN**

# Section F. HPLC Profile


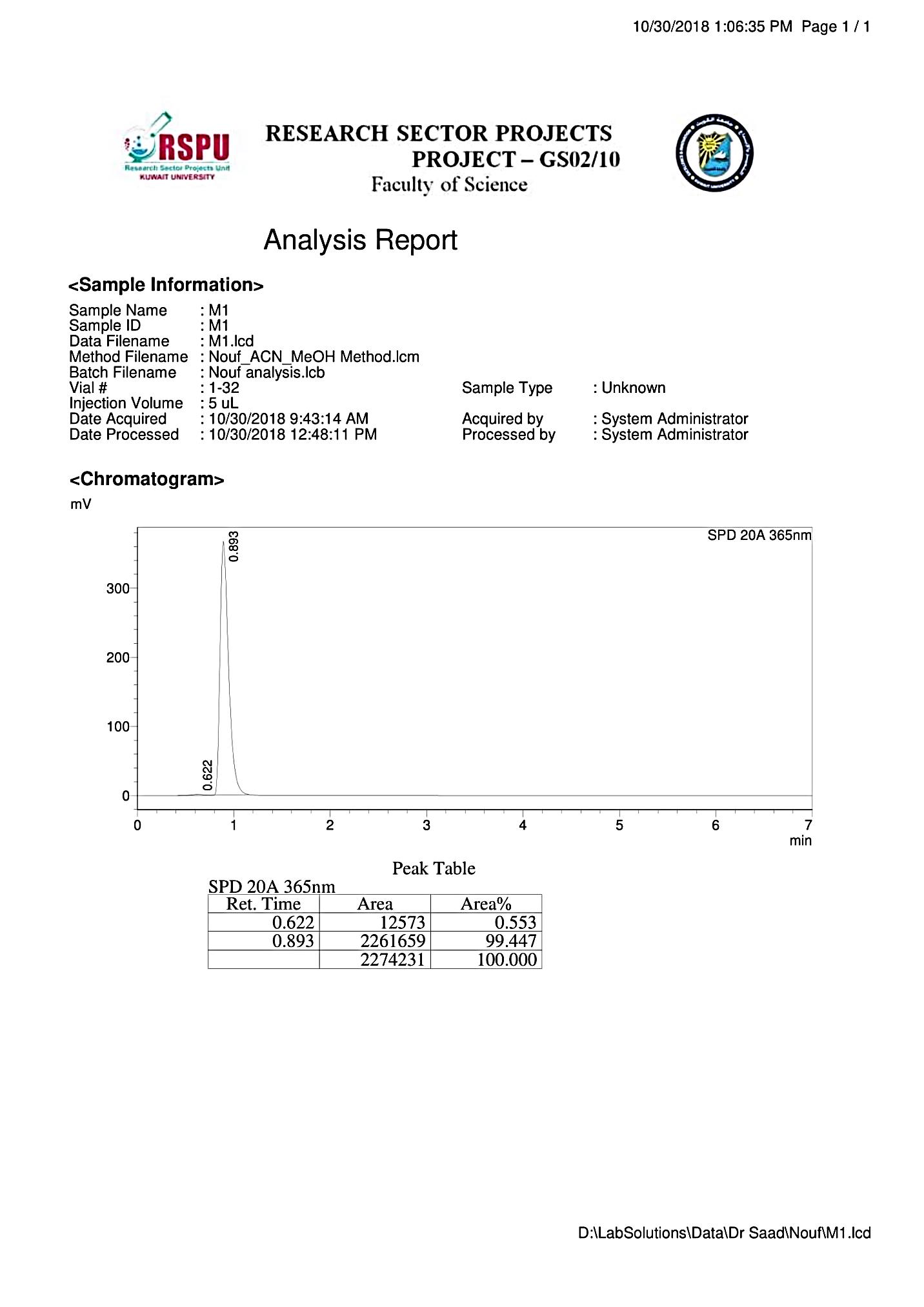


**Fig. S13. HPLC analysis of 2CzPN**


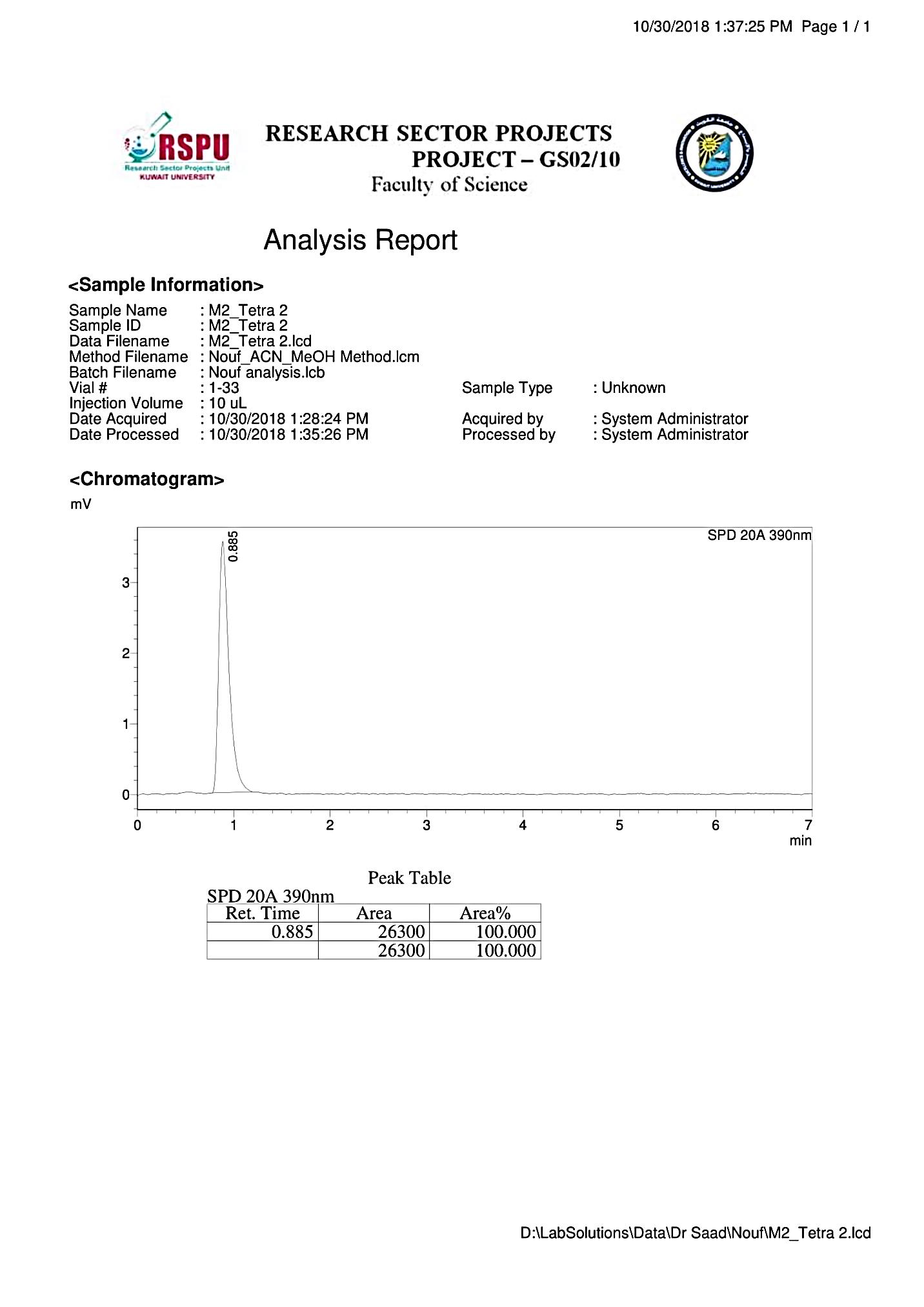


**Fig. S14. HPLC analysis of 4CzPN**

# Section G. DSC analysis of monomers

**Fig. S15. DSC analysis of monomers 2CzPN and 4CzPN**

# Section H. TGA Profiles

**Fig. S16. TGA Profiles of polymers p-2CzPN and p-4CzPN at a heating rate of 10 °C/min at nitrogen atmosphere**

# Section I. PXRD Profiles

**Fig. S17. Powder XRD Profiles of p-2CzPN and p-4CzPN with angular range between 10°- 80° and 2θ** **scan step of -0.015 by using CuKα source at 40kV voltage detected by Lynxeye detector**


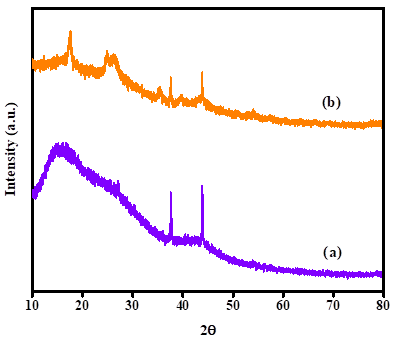


**Fig. S18. Powder XRD Profiles of (a) c-2CzPN-KOH and (b) c-4CzPN-KOH with angular range between 10°- 80° and 2θ** **scan step of -0.015 by using CuKα source at 40kV voltage detected by Lynxeye detector ^[3]^**

# Section J. Particle size analysis


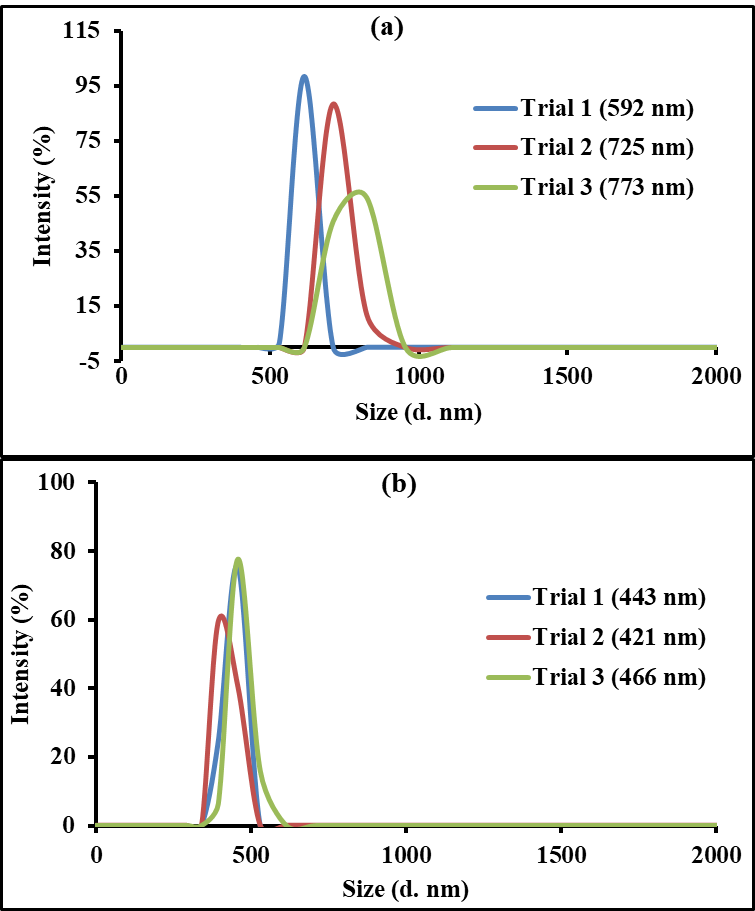


**Fig. S19. Particle size analysis of (a) (a) c-2CzPN-KOH and (b) c-4CzPN-KOH using Malvern Zeta sizer**

# Section K. XPS Profile


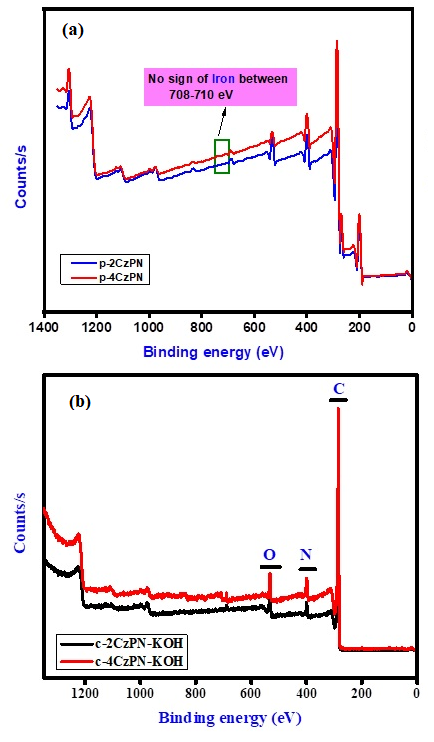


**Fig. S20. XPS Survey scan of (a) p-2CzPN and p-4CzPN polymer; (b) c-2CzPN-KOH and c-4CzPN-KOH using ESCALAB/AlKα source with spot size range from 200-850µm**

**Table S1** Elemental analysis of the prepared materials by XPS

| **Sample** | **Carbon** | | **Oxygen** | | **Nitrogen** | |
| --- | --- | --- | --- | --- | --- | --- |
|  | **Peak BE (eV)** | **Atomic**  **%** | **Peak BE (eV)** | **Atomic %** | **Peak BE (eV)** | **Atomic %** |
| **p-2CzPN** | 284.4 | 77.6 | 531.6 | 4.6 | 399.8 | 7.7 |
| **p-4CzPN** | 284.2 | 80.1 | 532.2 | 2.3 | 399.9 | 7.2 |
| **c-2CzPN-KOH** | 284.6 | 89.2 | 531.5 | 7.1 | 399.8 | 3.7 |
| **c-4CzPN-KOH** | 284.6 | 90.2 | 532.1 | 4.6 | 398.4 | 4.9 |

# Section L. SEM-EDX analysis of the carbon materials


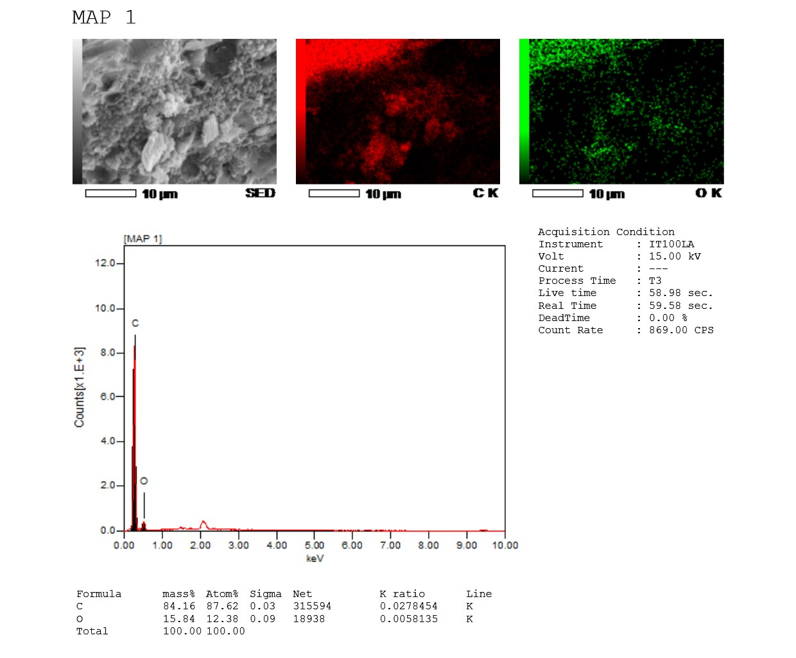


**Fig. S21. EDX elemental mapping of c-2CzPN-KOH**


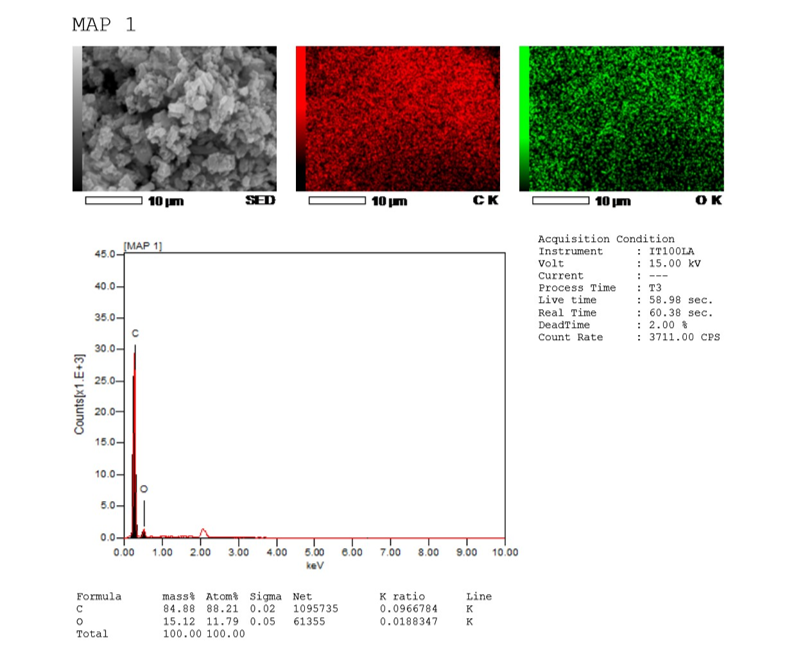


**Fig. S22. EDX elemental mapping of c-4CzPN-KOH**

#

# Section M. CO_2_/N_2_ Selectivity studies


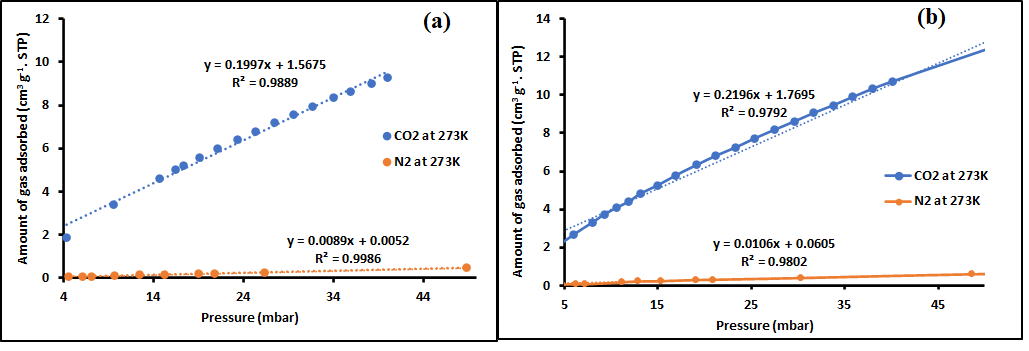


**Fig. S23. CO_2_/N_2_ adsorption selectivity of (a) c-2CzPN-KOH and (b) c-2CzPN-KOH calculated using Henry’s law initial slope method at 273K**


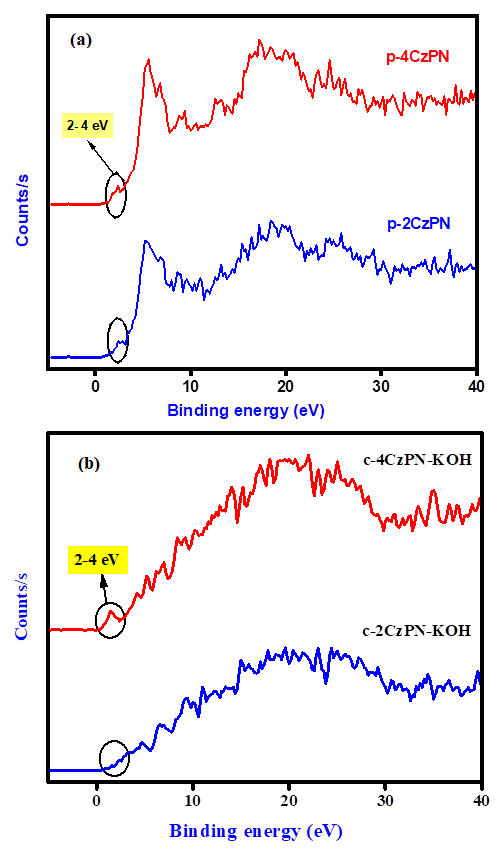


**Fig. S24. Valence band spectrum of (a) p-2CzPN and p-4CzPN polymer; (b) c-2CzPN-KOH and c-4CzPN-KOH**

# References

1. T. V. Nagalakshmi, K. A. Emmanuel, Ch. Suresh Babu, Ch. Chakrapani, P. Paul Divakar. Preparation Of Mesoporous Activated Carbon from Jackfruit Ppi-1 Waste and Development Of Different Surface Functional Groups. International Letters of Chemistry, Physics and Astronomy, V54,189-200.
2. Dingding Yao, Yeshui Zhang, Paul T. Williamsb, Haiping Yanga, Hanping Chen. Co-production of hydrogen and carbon nanotubes from real-world waste plastics: Influence of catalyst composition and operational parameters, Applied Catalysis B: Environmental 221 (2018) 584–597.
3. Thanapat Autthawong, Orapim Namsar, Aishui Yu, Thapanee Sarakonsri. Cost‑efective production of SiO2/C and Si/C composites derived from rice husk for advanced lithium‑ion battery anodes. Journal of Materials Science: Materials in Electronics, 2020.
